# Supplementary figures and images for: A fine-tuned YOLOv5 deep learning approach for real-time house number detection
Source: PeerJ Comput Sci. 2023 Jul 3;9:e1453. doi: 10.7717/peerj-cs.1453 (PMC10403189; doi:10.7717/peerj-cs.1453)

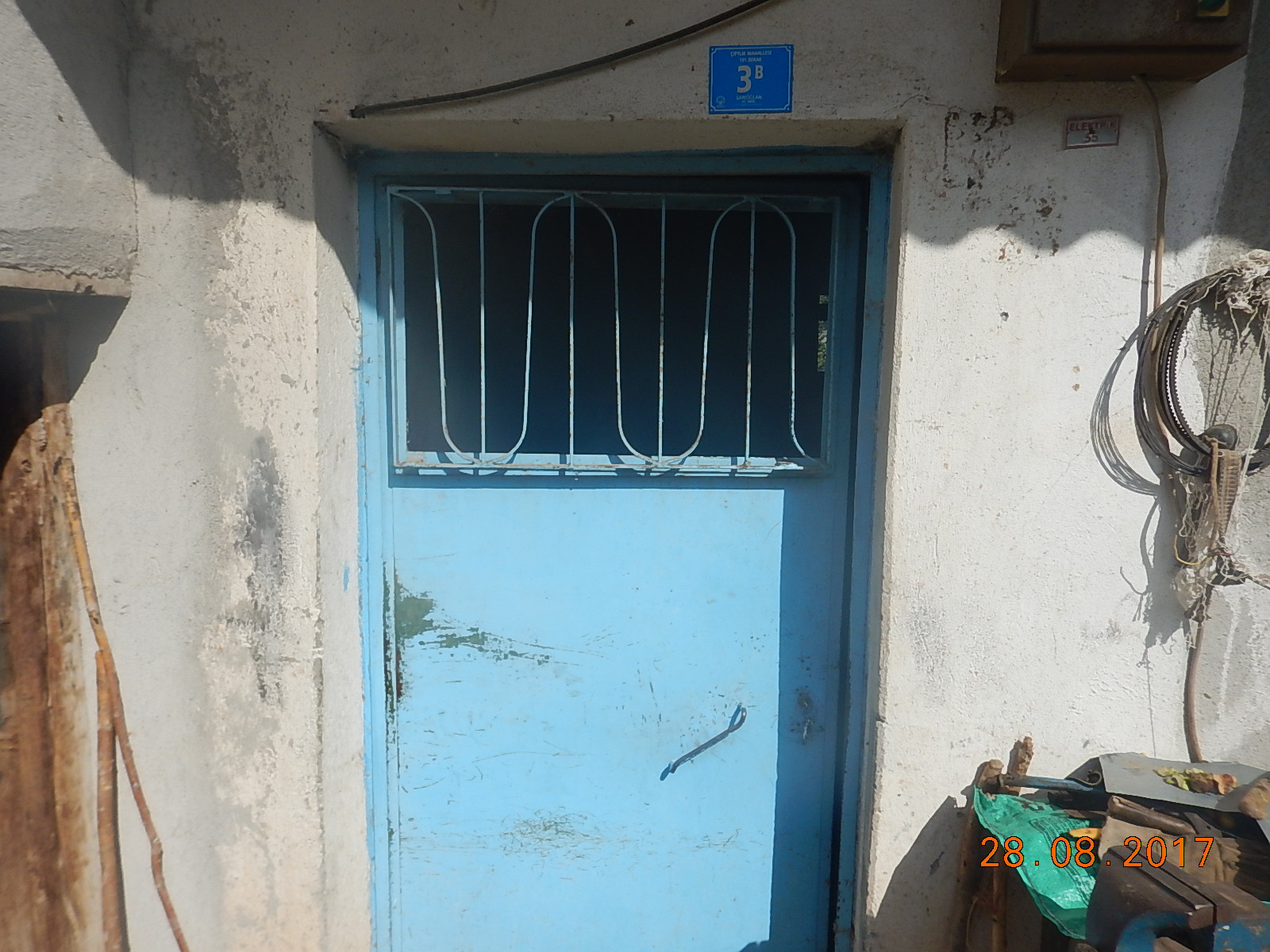

Supplement: Supplemental Information 2 [file peerj-cs-09-1453-s002.zip › ExampleDataFile/10xxyyyz3B.JPG]

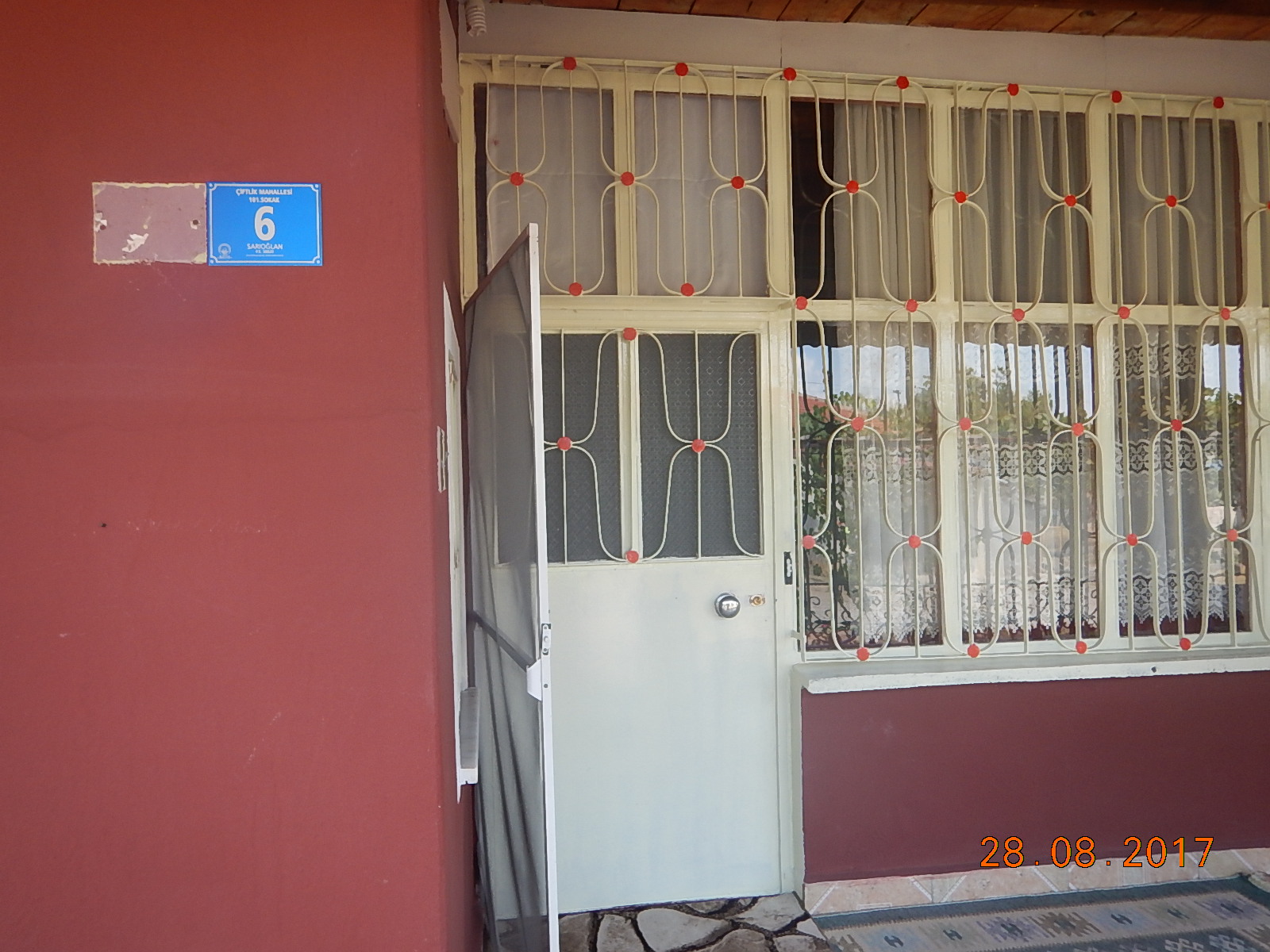

Supplement: Supplemental Information 2 [file peerj-cs-09-1453-s002.zip › ExampleDataFile/11xxyyyz6.JPG]

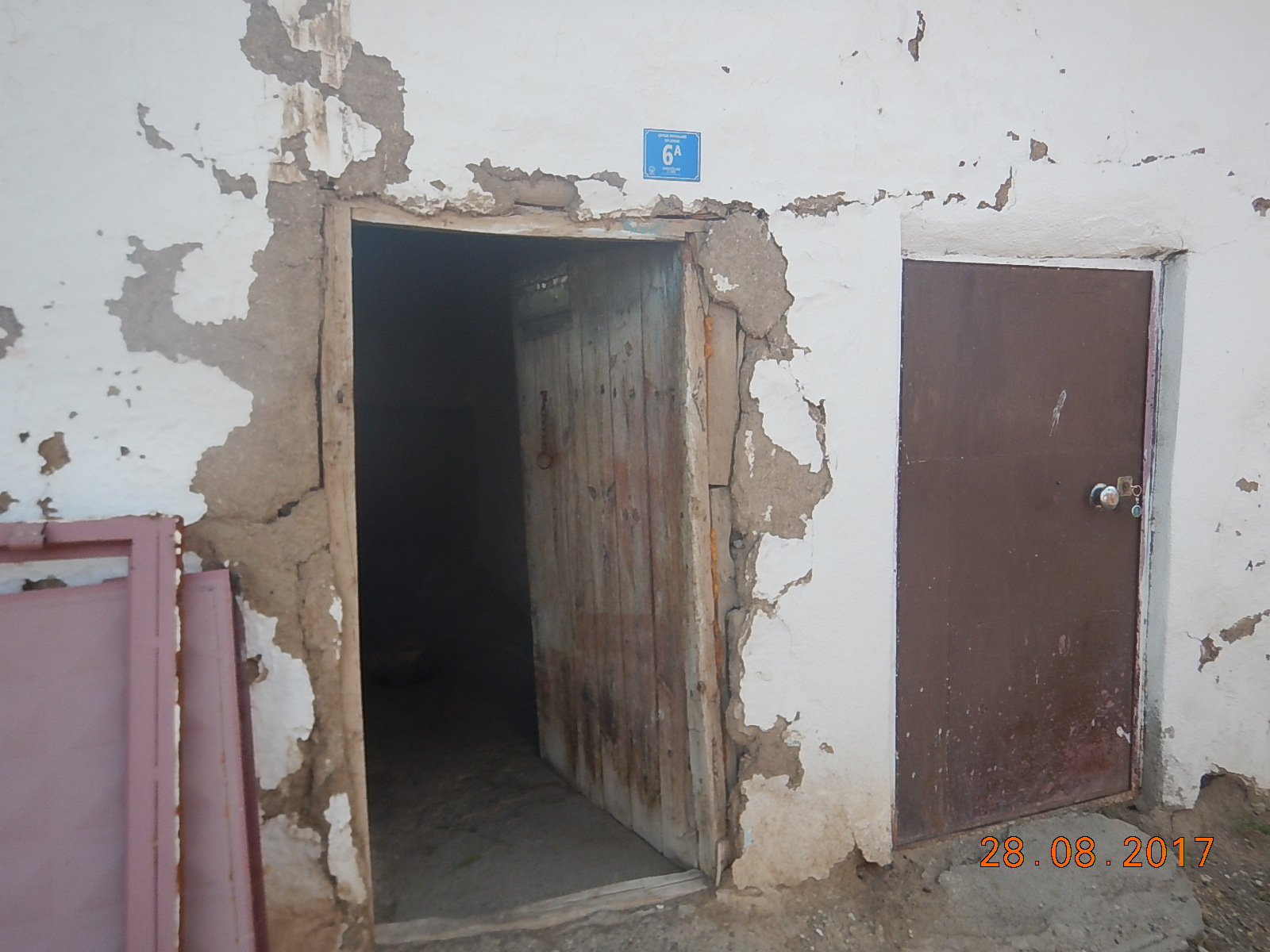

Supplement: Supplemental Information 2 [file peerj-cs-09-1453-s002.zip › ExampleDataFile/12xxyyyz6A.JPG]

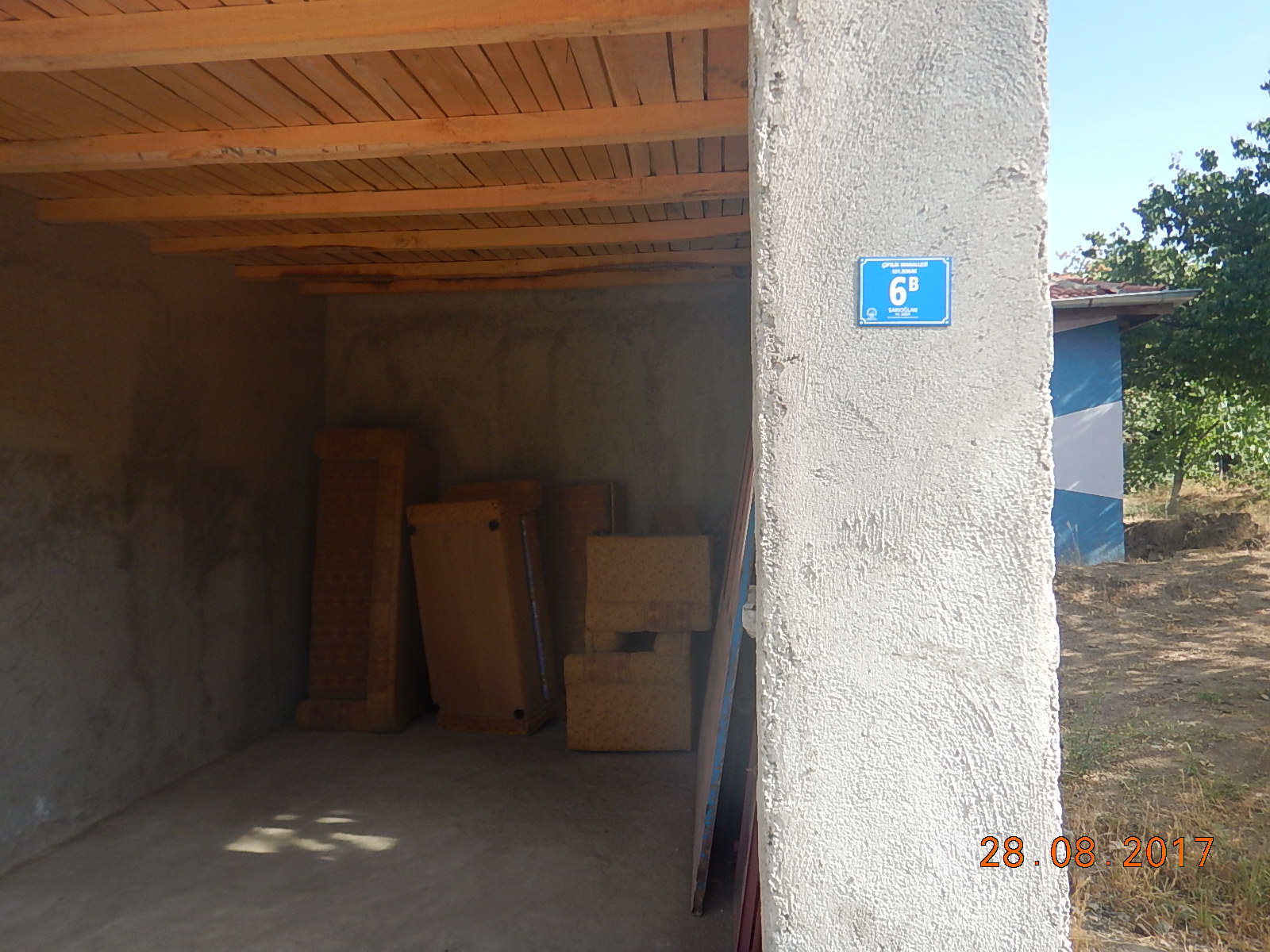

Supplement: Supplemental Information 2 [file peerj-cs-09-1453-s002.zip › ExampleDataFile/13xxyyyz6B.JPG]

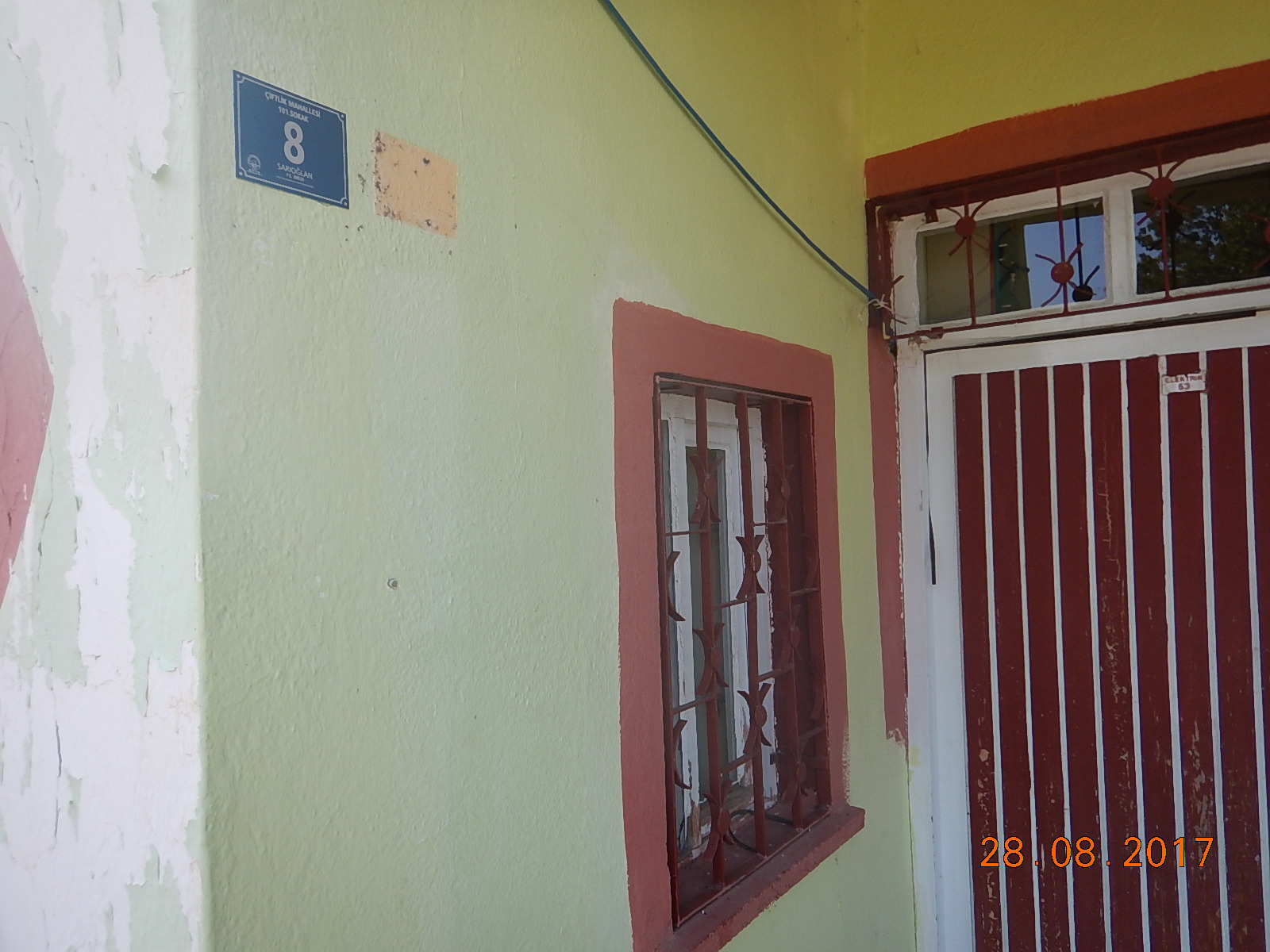

Supplement: Supplemental Information 2 [file peerj-cs-09-1453-s002.zip › ExampleDataFile/14xxyyyz8.JPG]

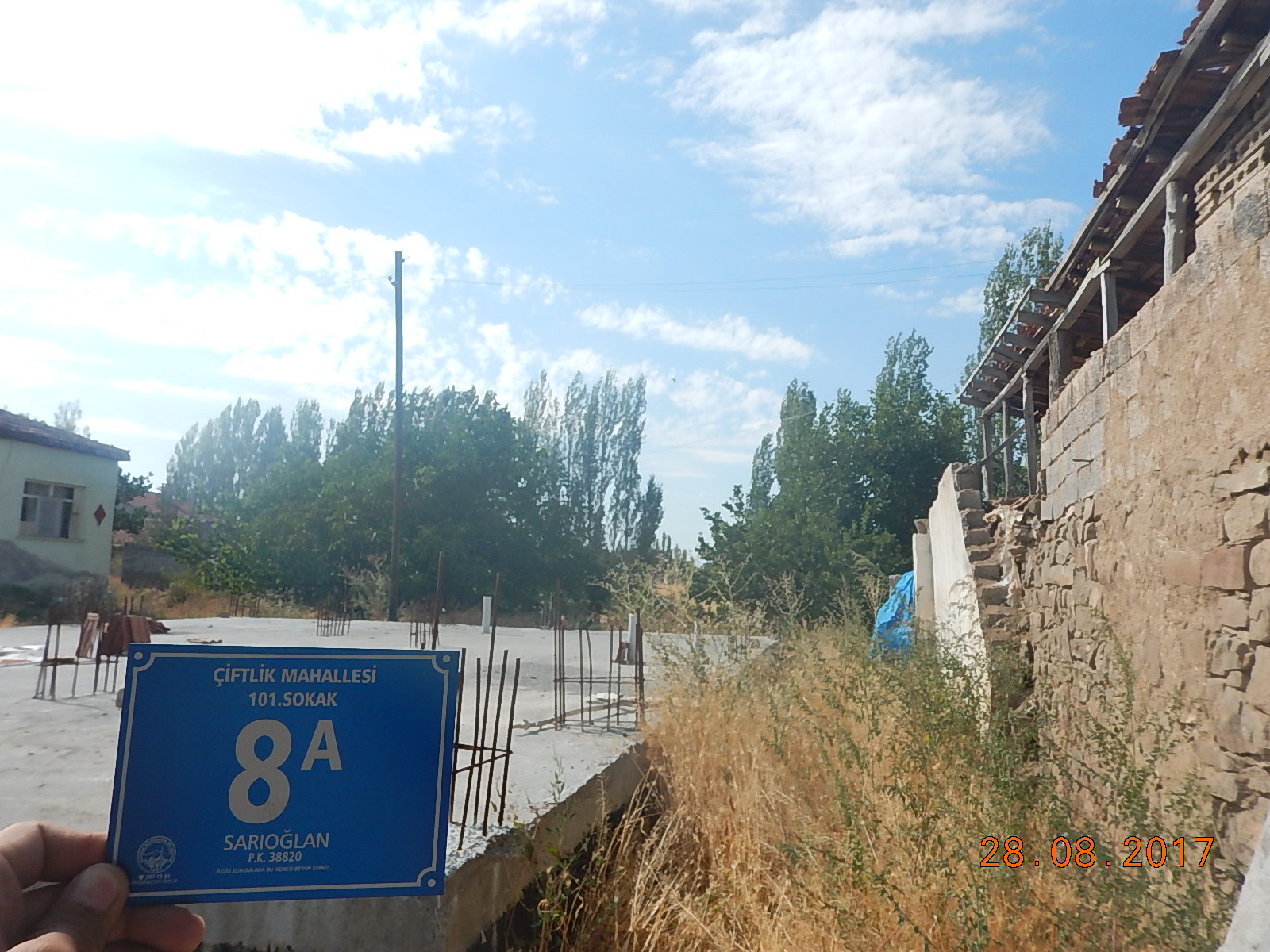

Supplement: Supplemental Information 2 [file peerj-cs-09-1453-s002.zip › ExampleDataFile/15xxyyyz8A.JPG]

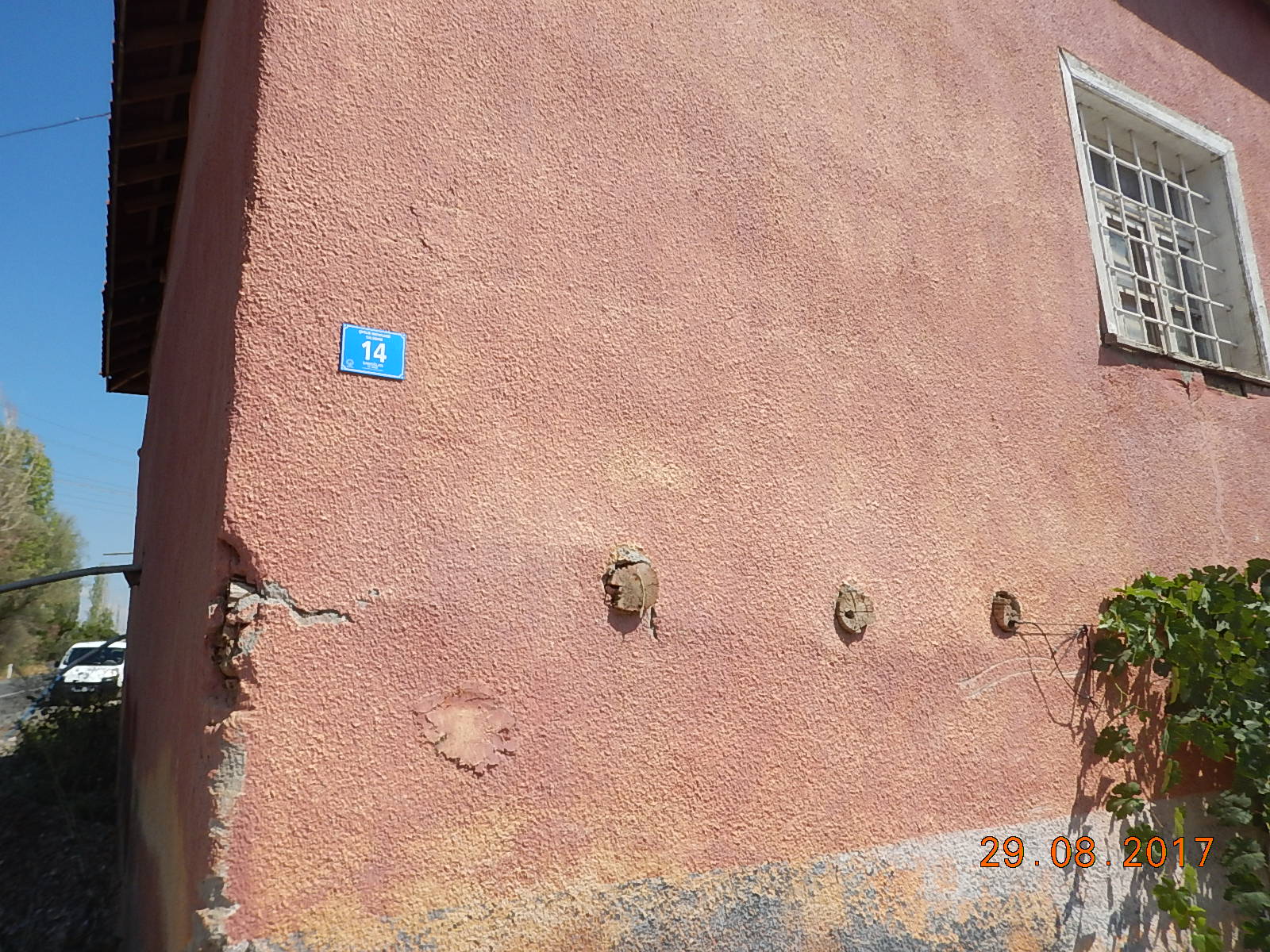

Supplement: Supplemental Information 2 [file peerj-cs-09-1453-s002.zip › ExampleDataFile/16xxyyyz14.JPG]

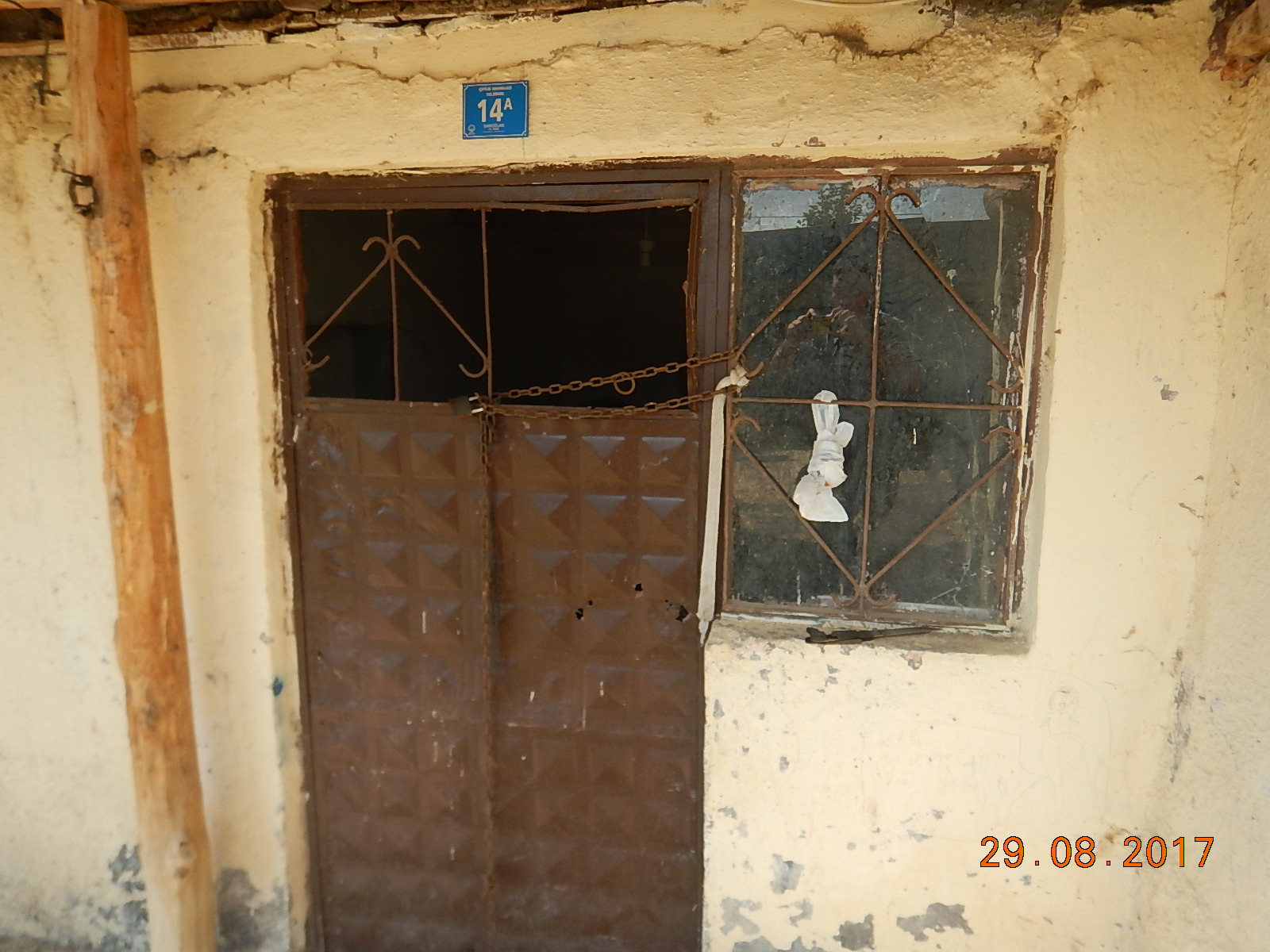

Supplement: Supplemental Information 2 [file peerj-cs-09-1453-s002.zip › ExampleDataFile/17xxyyyz14A.JPG]

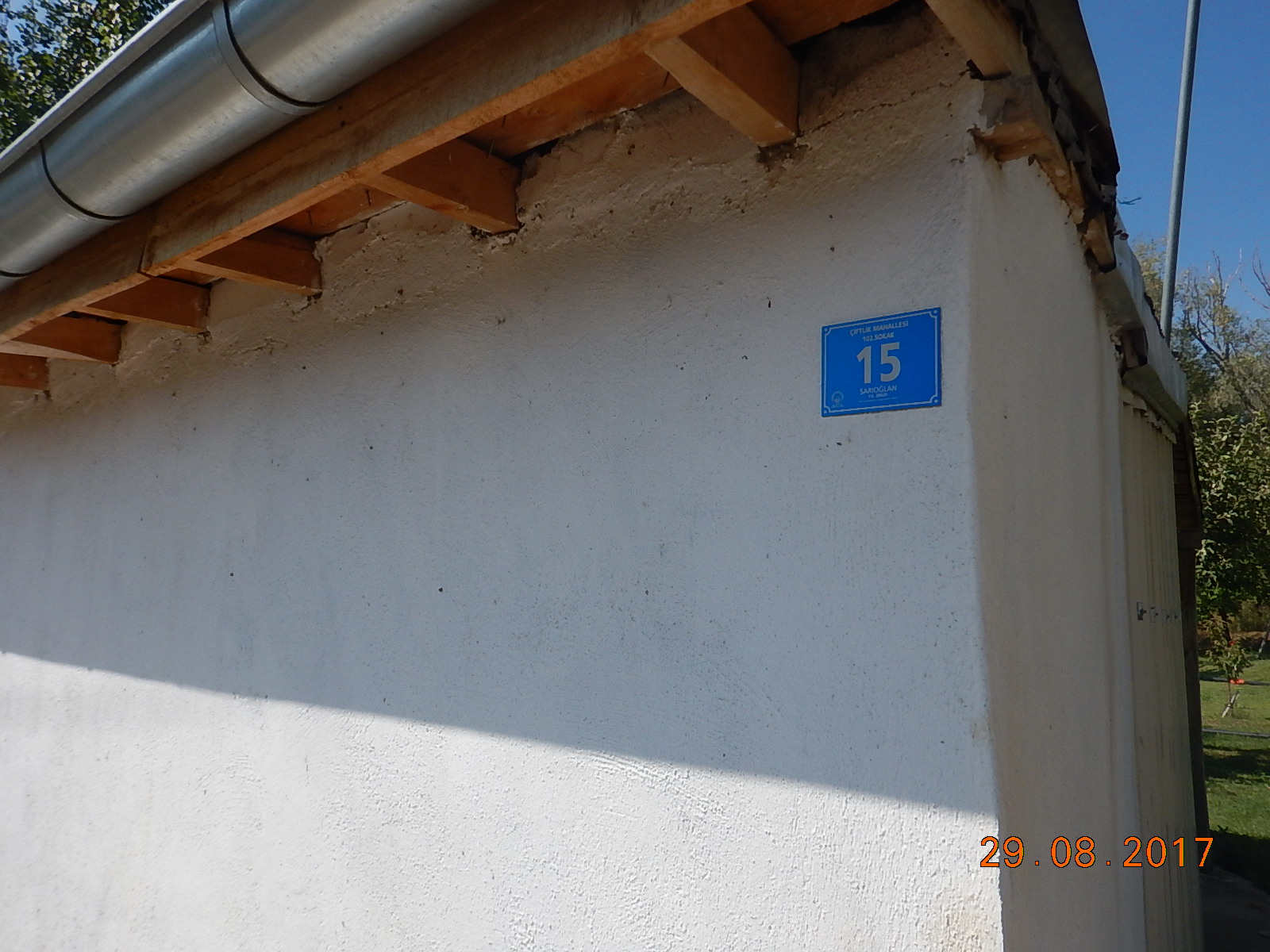

Supplement: Supplemental Information 2 [file peerj-cs-09-1453-s002.zip › ExampleDataFile/18xxyyyz15.JPG]

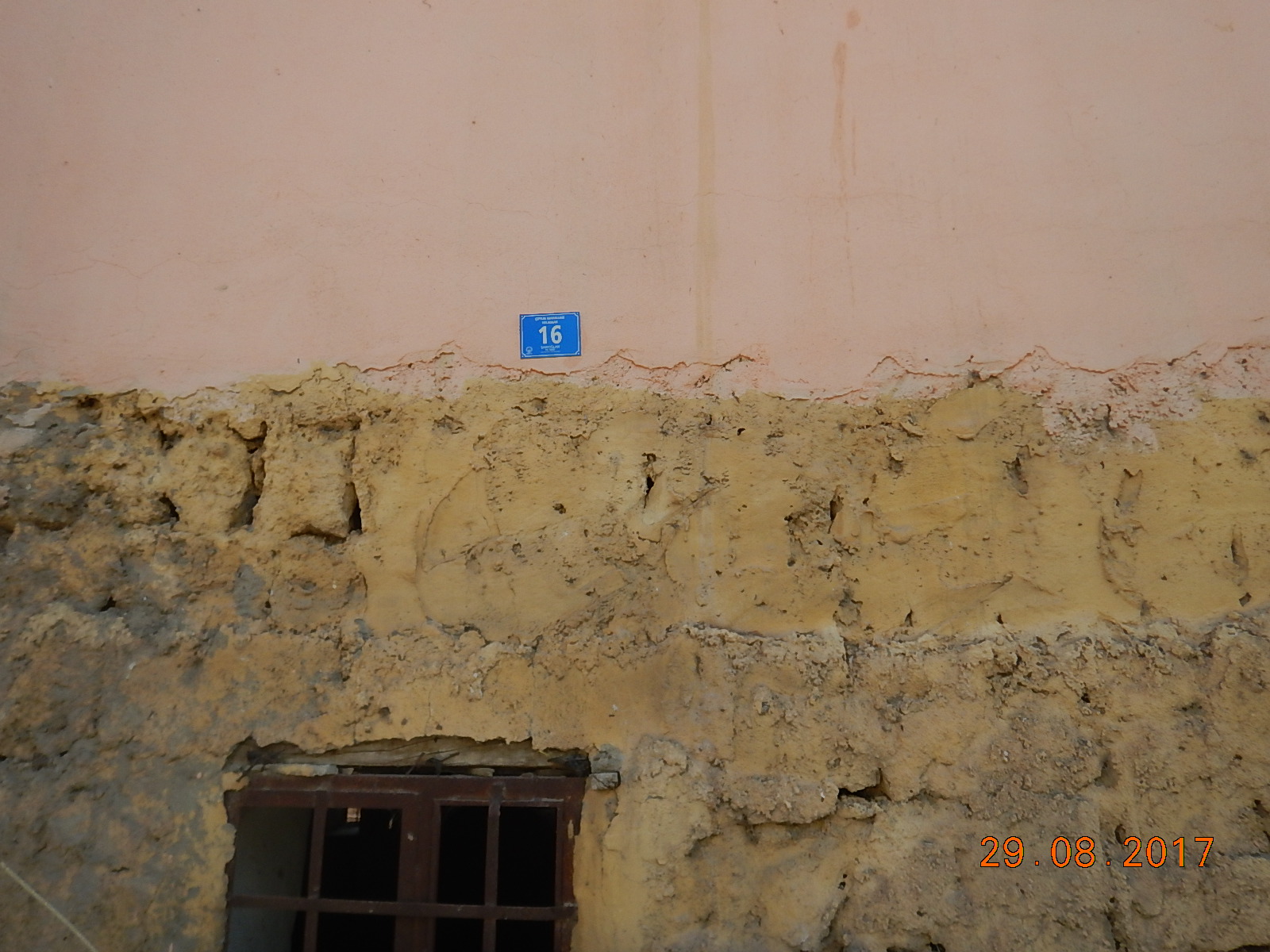

Supplement: Supplemental Information 2 [file peerj-cs-09-1453-s002.zip › ExampleDataFile/19xxyyyz16.JPG]

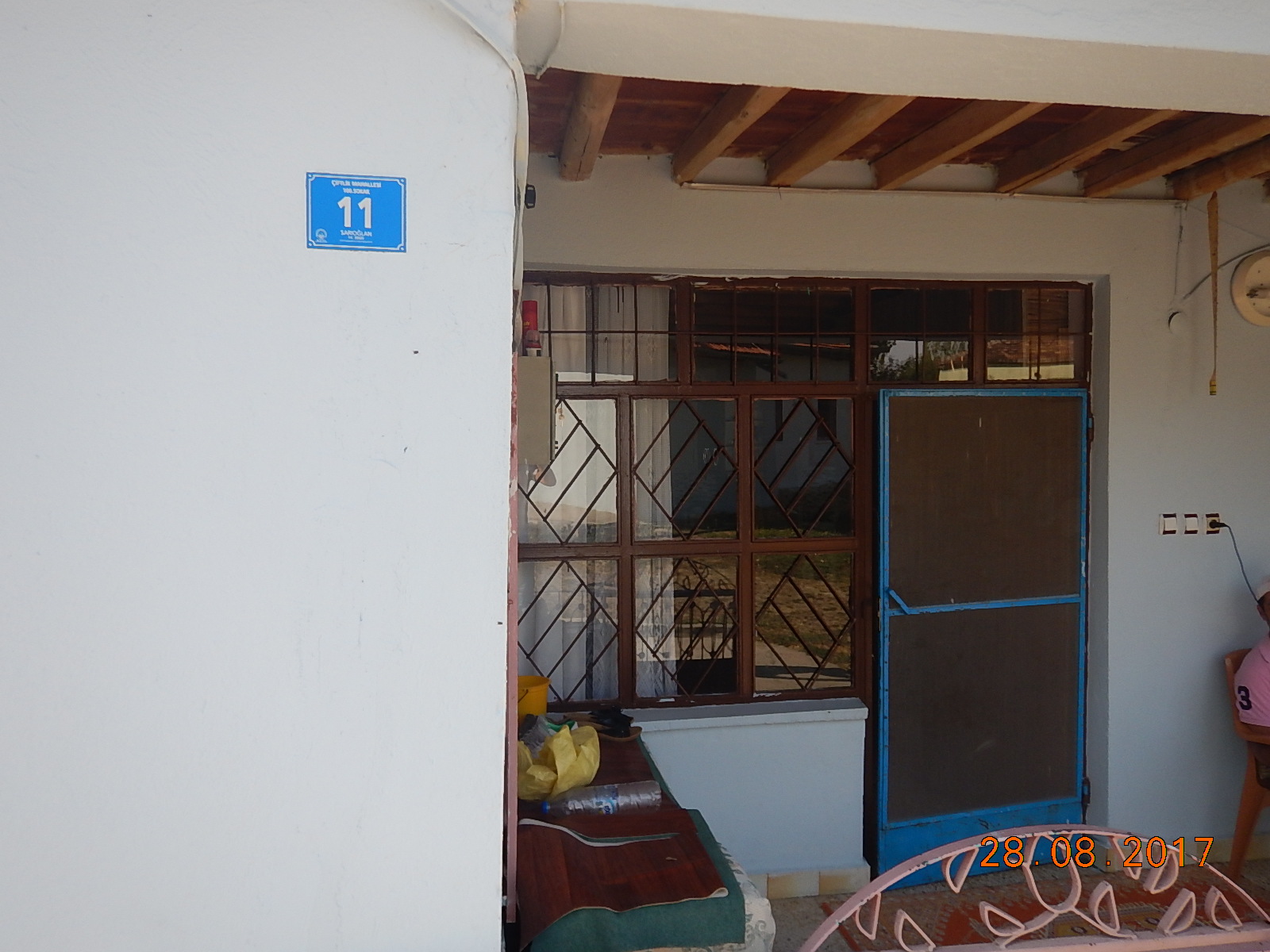

Supplement: Supplemental Information 2 [file peerj-cs-09-1453-s002.zip › ExampleDataFile/1xxyyyz11.JPG]

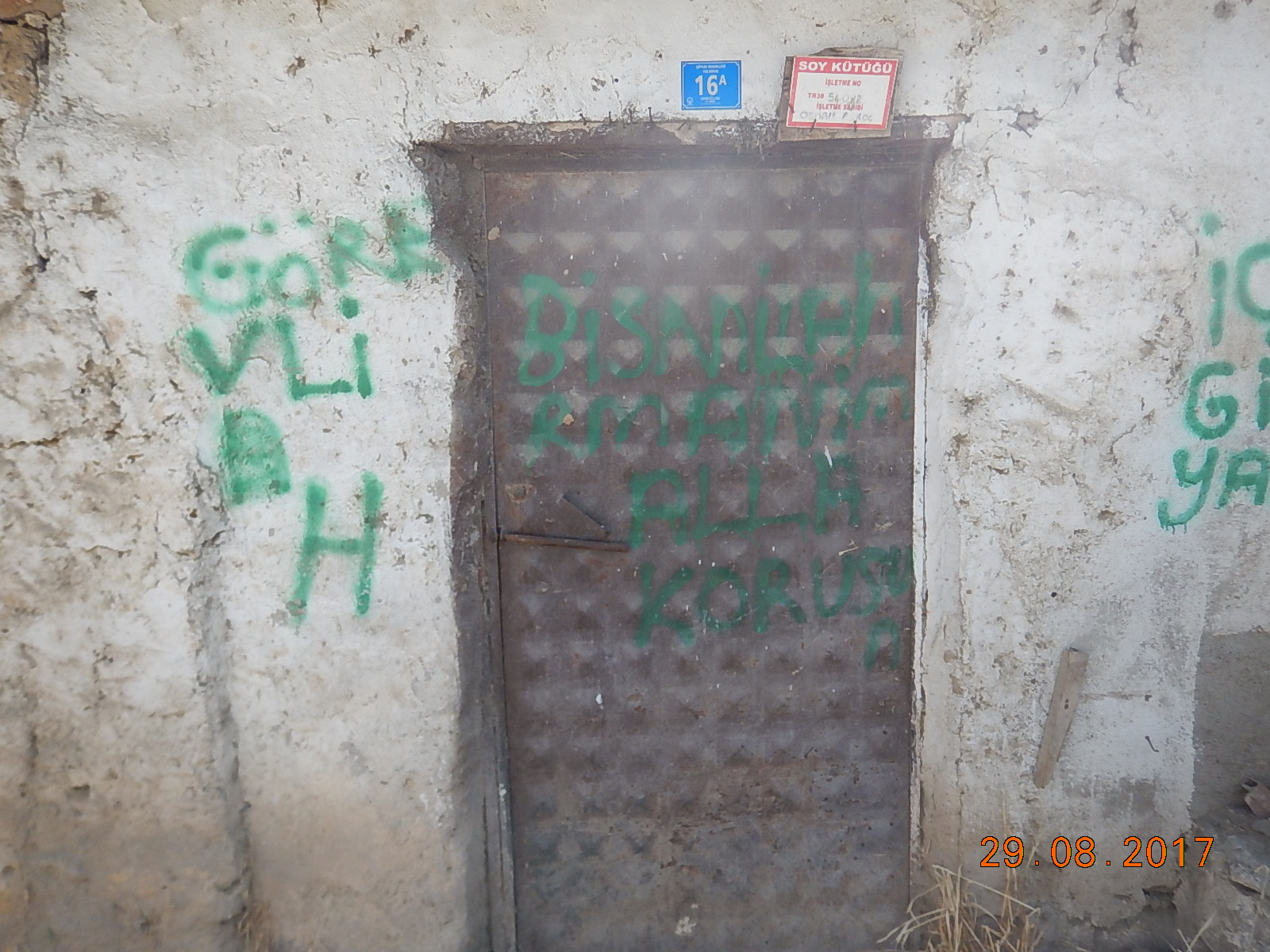

Supplement: Supplemental Information 2 [file peerj-cs-09-1453-s002.zip › ExampleDataFile/20xxyyyz16A.JPG]

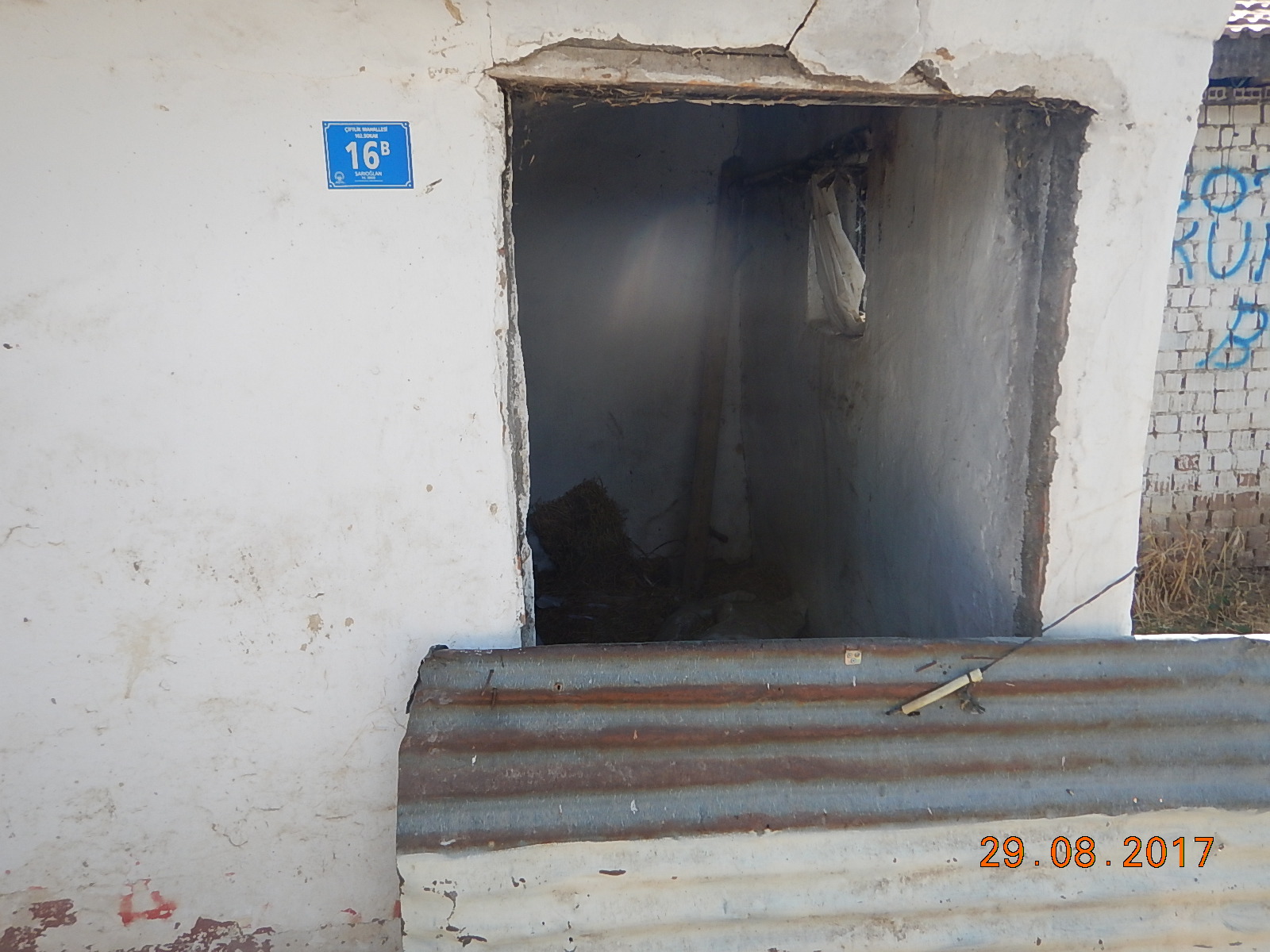

Supplement: Supplemental Information 2 [file peerj-cs-09-1453-s002.zip › ExampleDataFile/21xxyyyz16B.JPG]

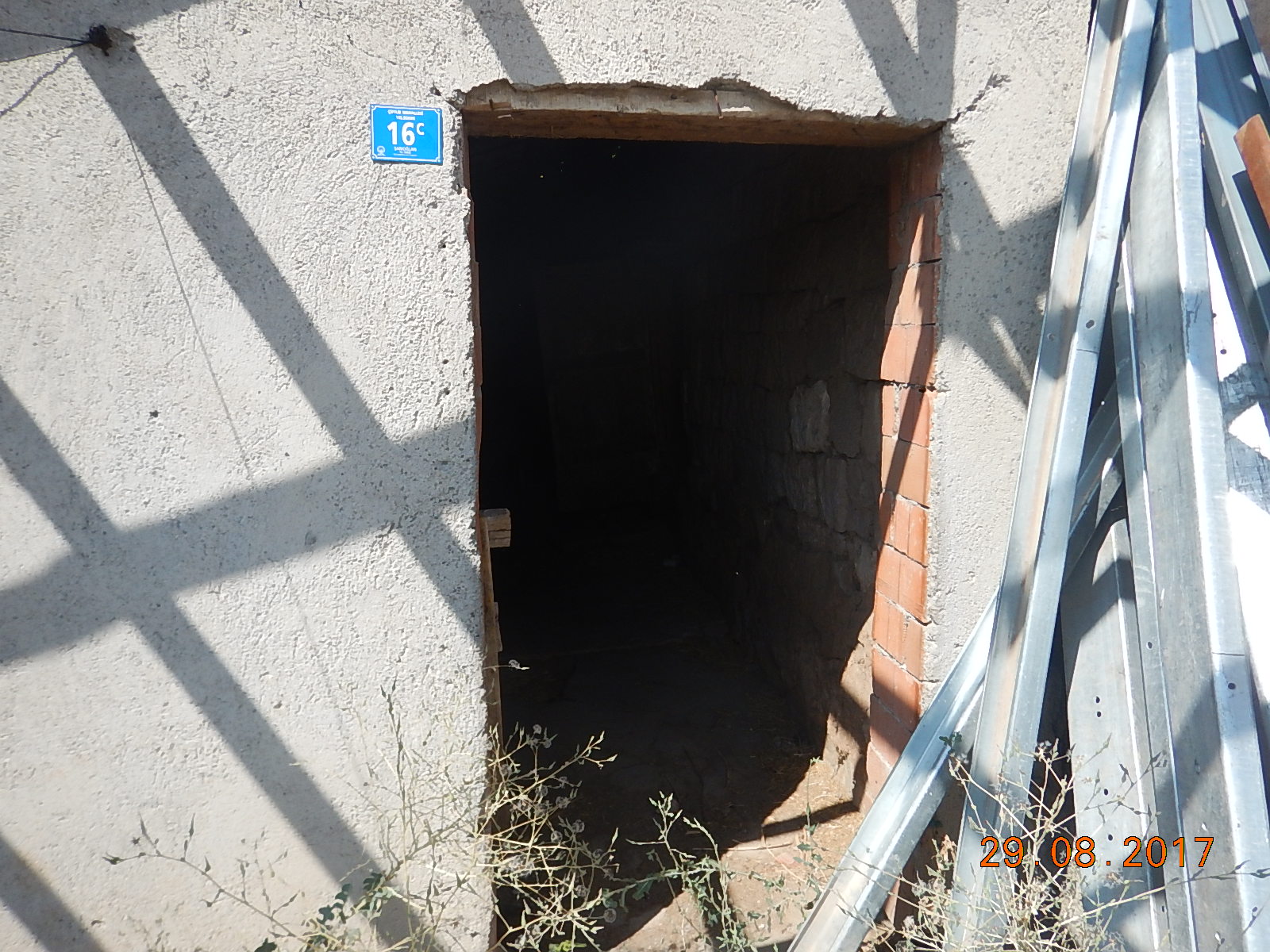

Supplement: Supplemental Information 2 [file peerj-cs-09-1453-s002.zip › ExampleDataFile/22xxyyyz16C.JPG]

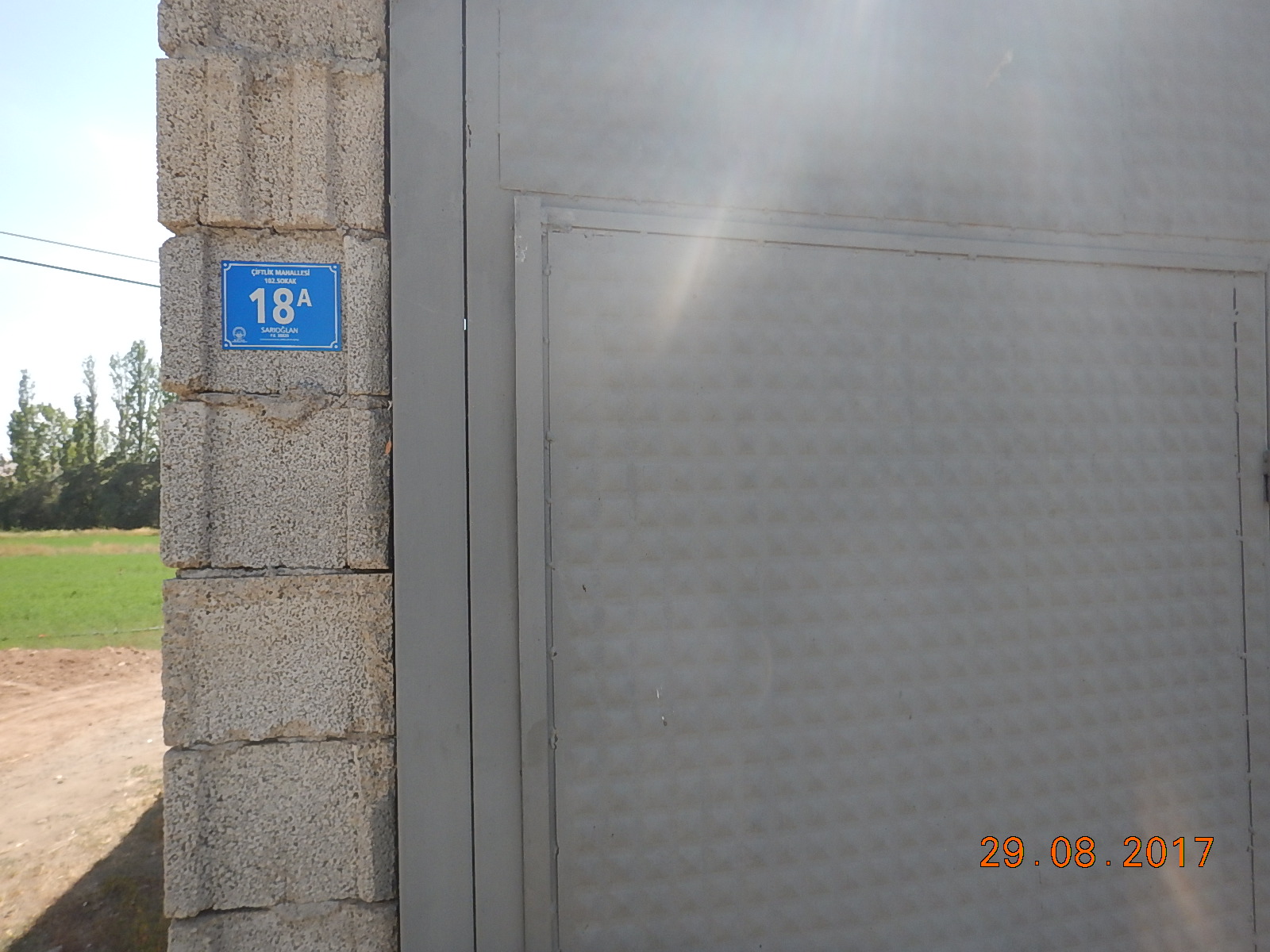

Supplement: Supplemental Information 2 [file peerj-cs-09-1453-s002.zip › ExampleDataFile/23xxyyyz18A.JPG]

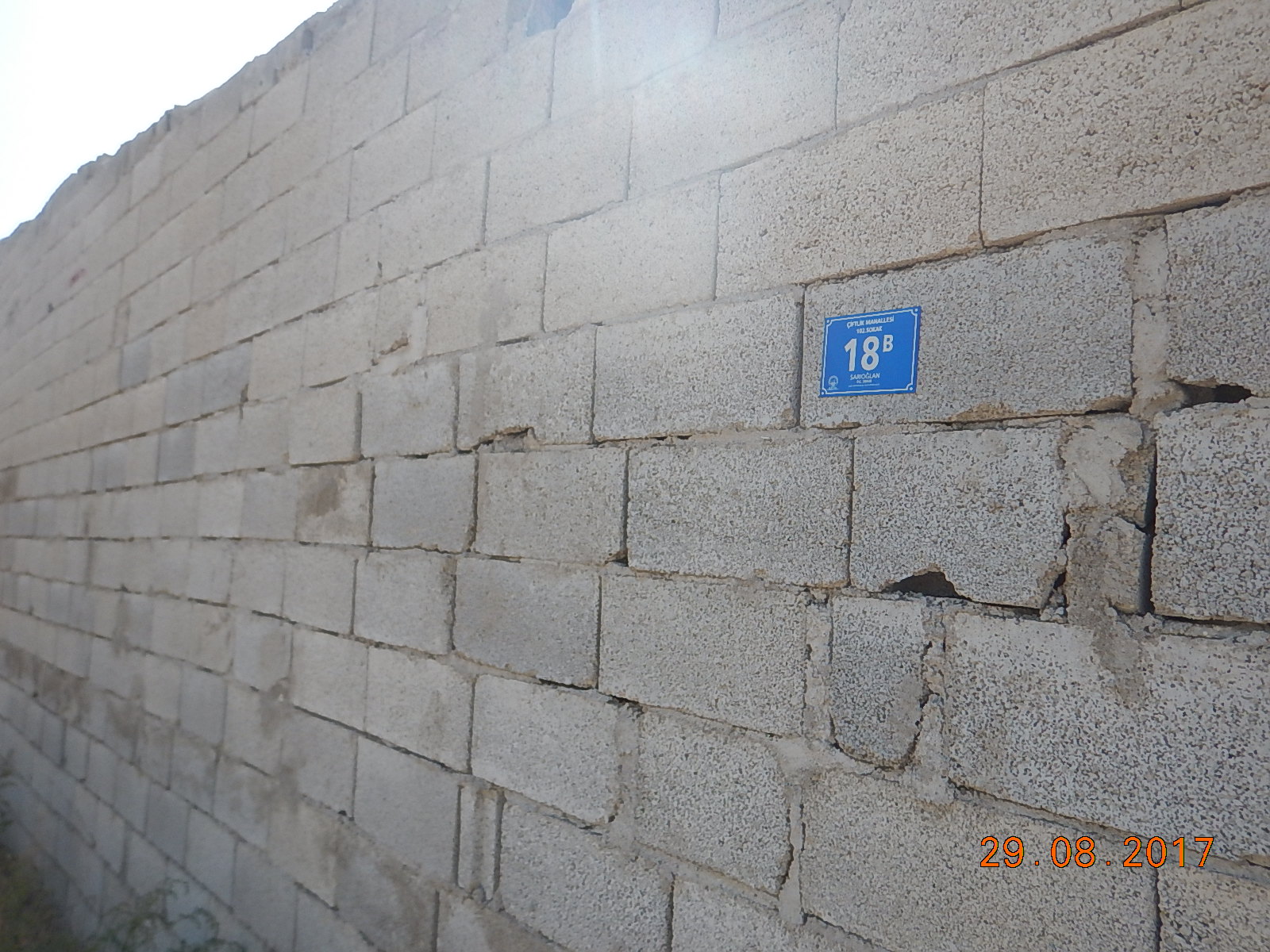

Supplement: Supplemental Information 2 [file peerj-cs-09-1453-s002.zip › ExampleDataFile/24xxyyyz18B.JPG]

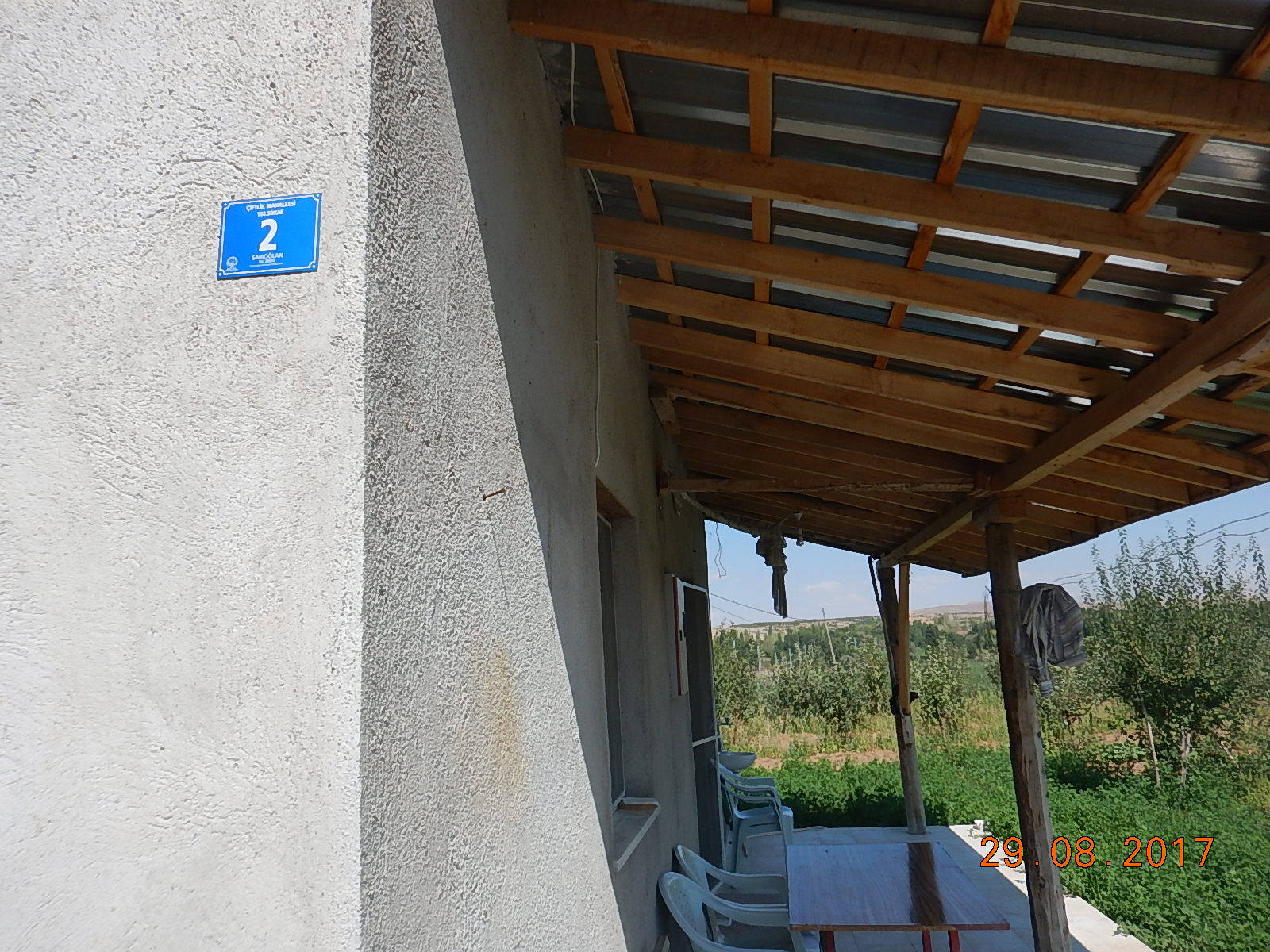

Supplement: Supplemental Information 2 [file peerj-cs-09-1453-s002.zip › ExampleDataFile/25xxyyyz2.JPG]

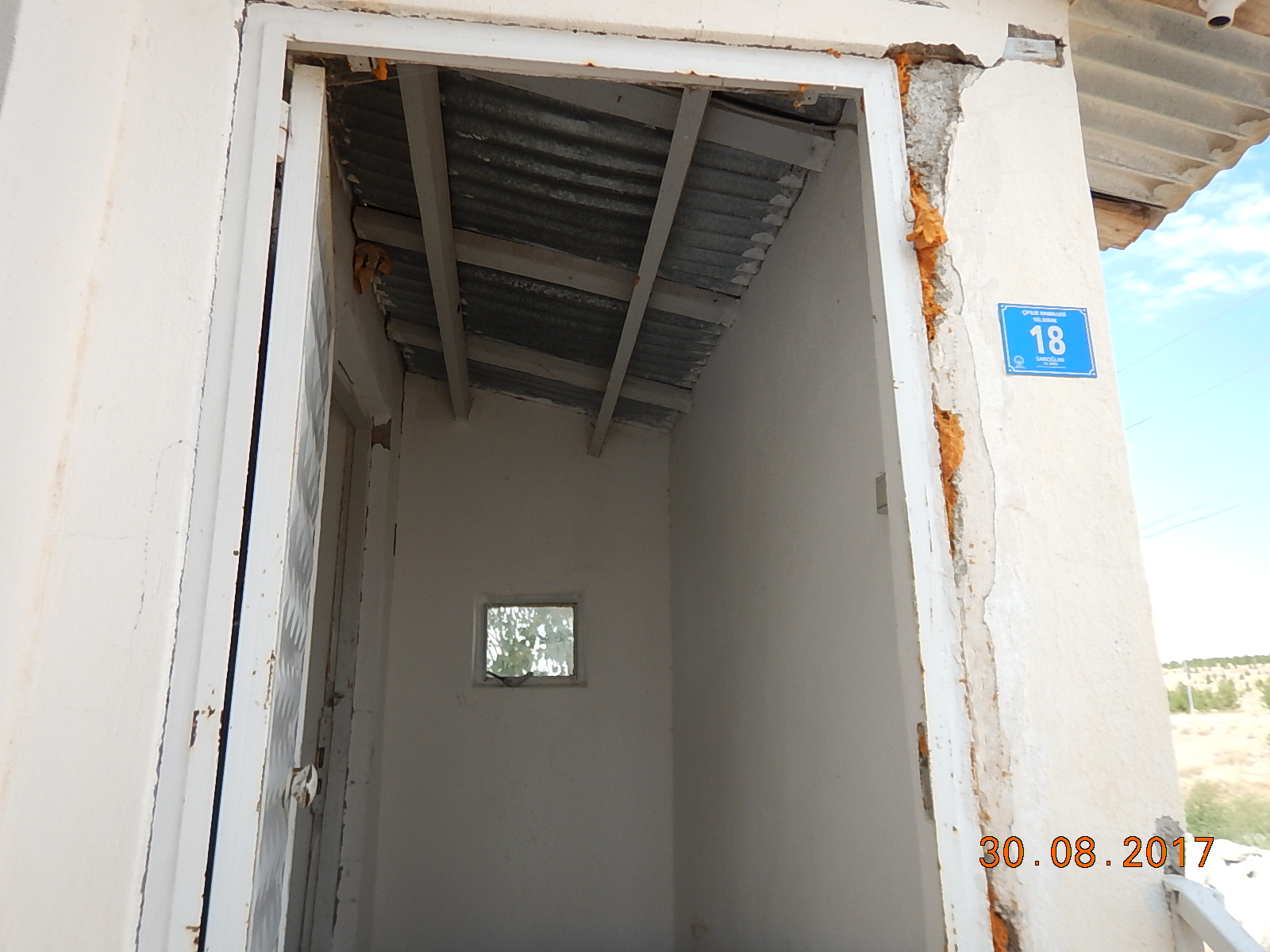

Supplement: Supplemental Information 2 [file peerj-cs-09-1453-s002.zip › ExampleDataFile/26xxyyyz18.JPG]

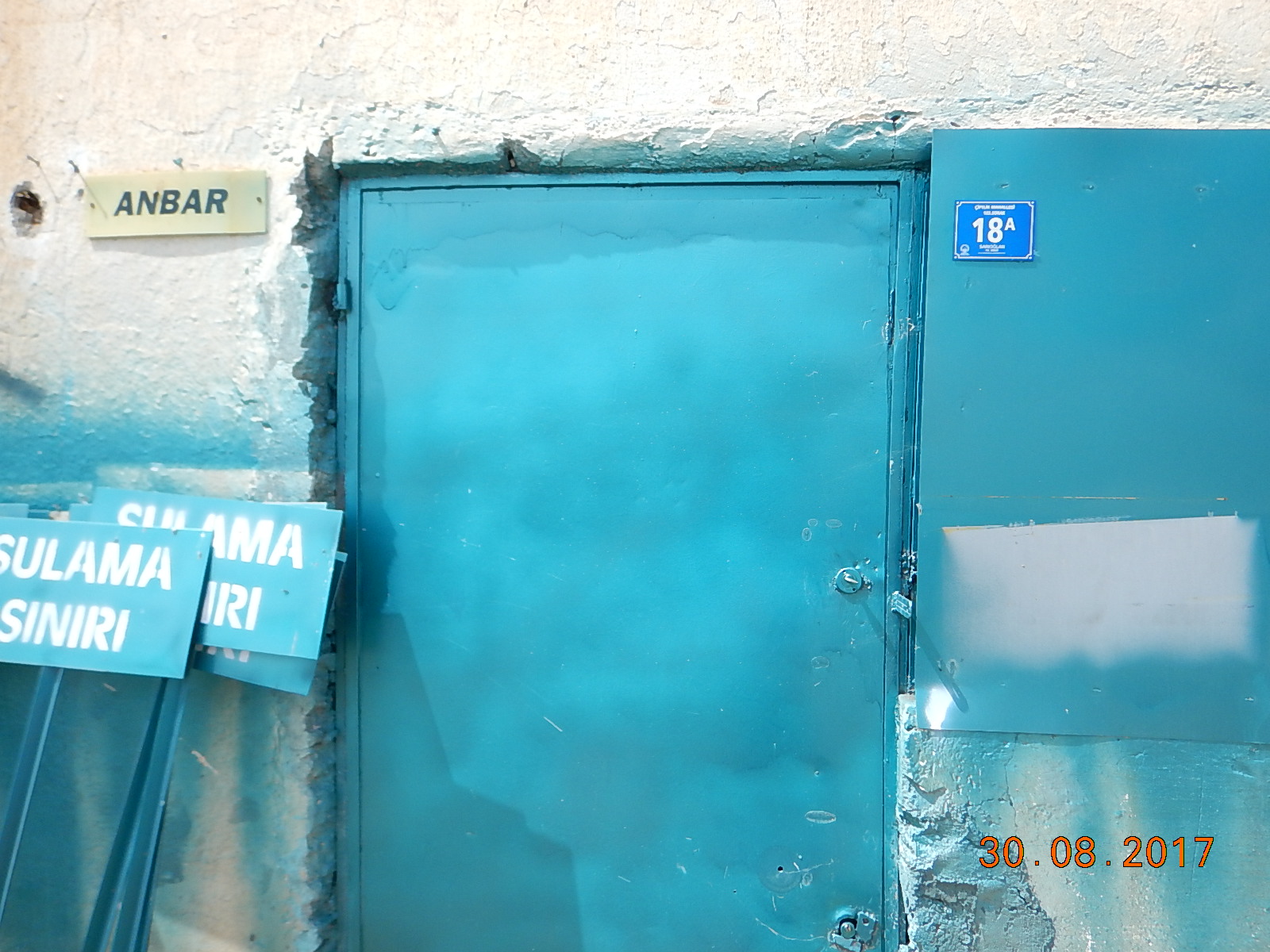

Supplement: Supplemental Information 2 [file peerj-cs-09-1453-s002.zip › ExampleDataFile/27xxyyyz18A.JPG]

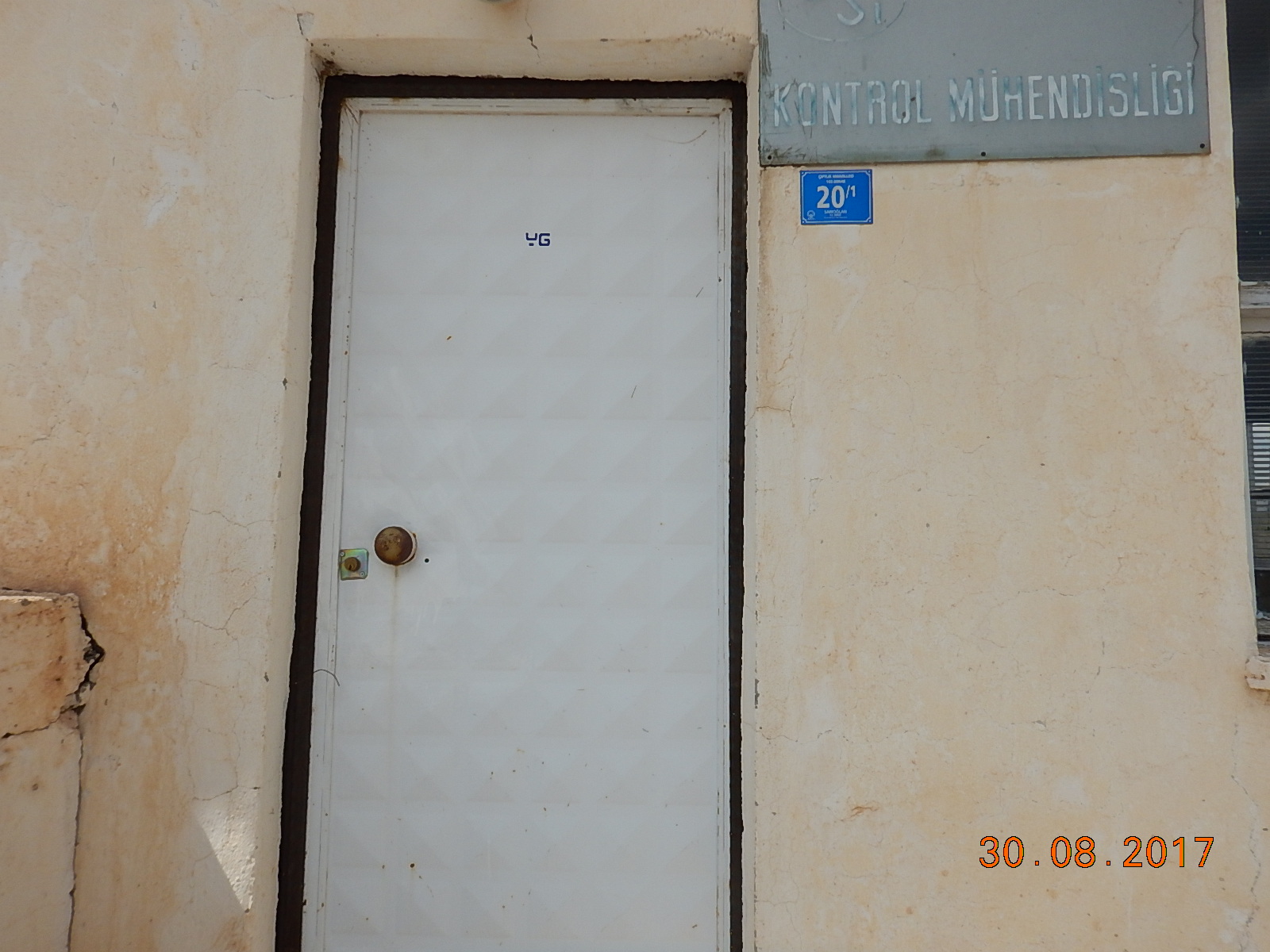

Supplement: Supplemental Information 2 [file peerj-cs-09-1453-s002.zip › ExampleDataFile/28xxyyyz20.1.JPG]

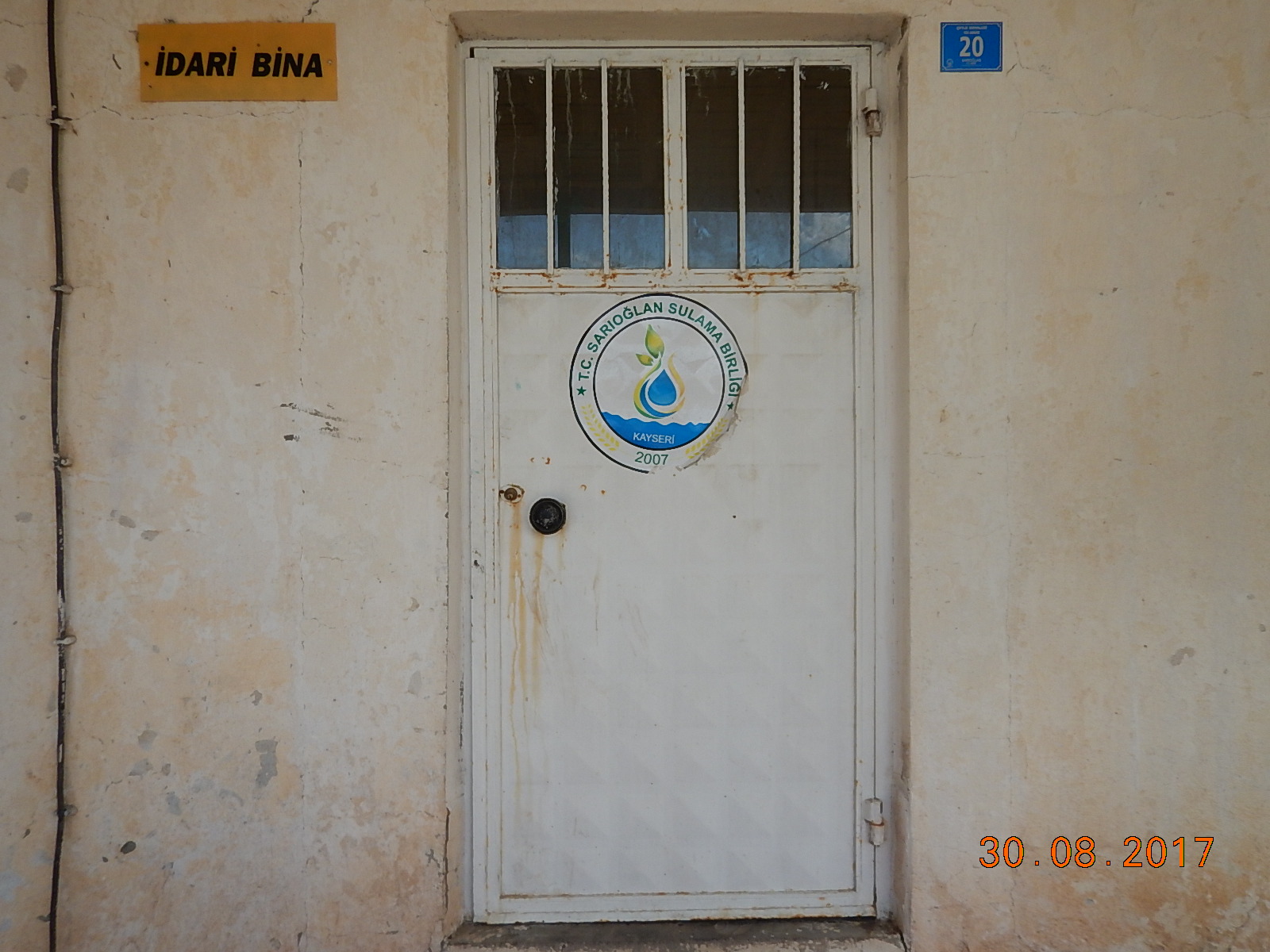

Supplement: Supplemental Information 2 [file peerj-cs-09-1453-s002.zip › ExampleDataFile/29xxyyyz20.JPG]

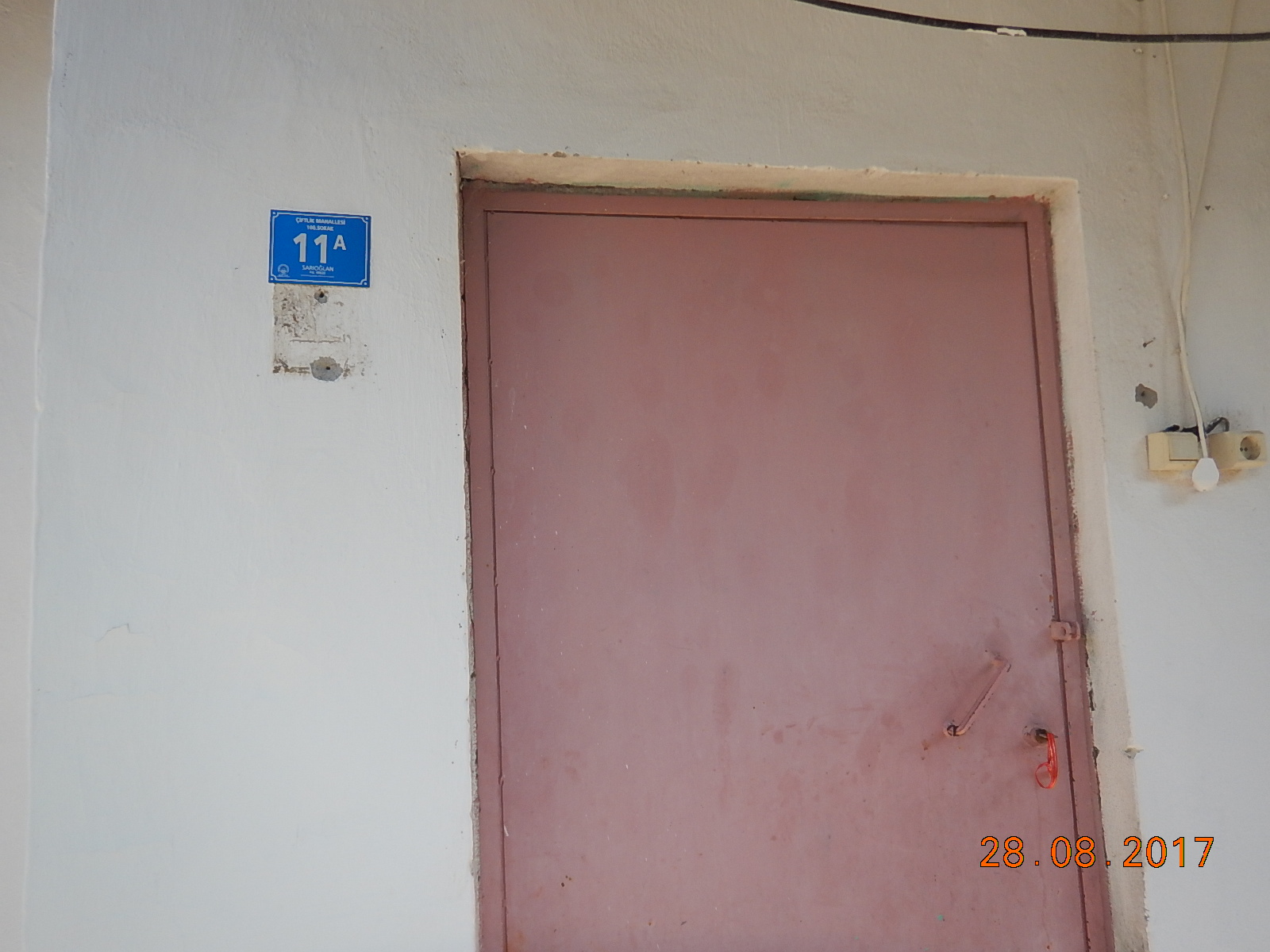

Supplement: Supplemental Information 2 [file peerj-cs-09-1453-s002.zip › ExampleDataFile/2xxyyyz11A.JPG]

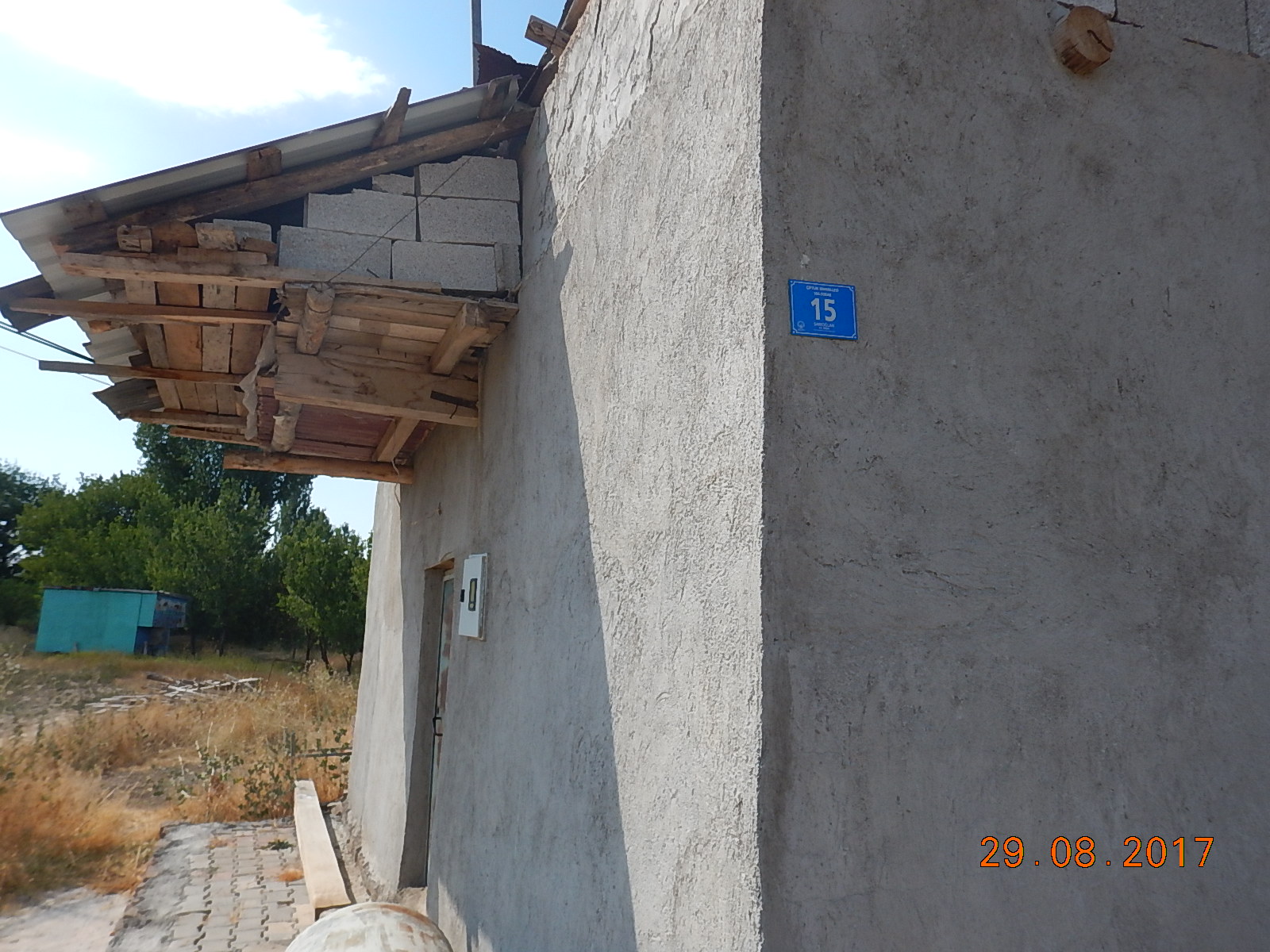

Supplement: Supplemental Information 2 [file peerj-cs-09-1453-s002.zip › ExampleDataFile/30xxyyyz15.JPG]

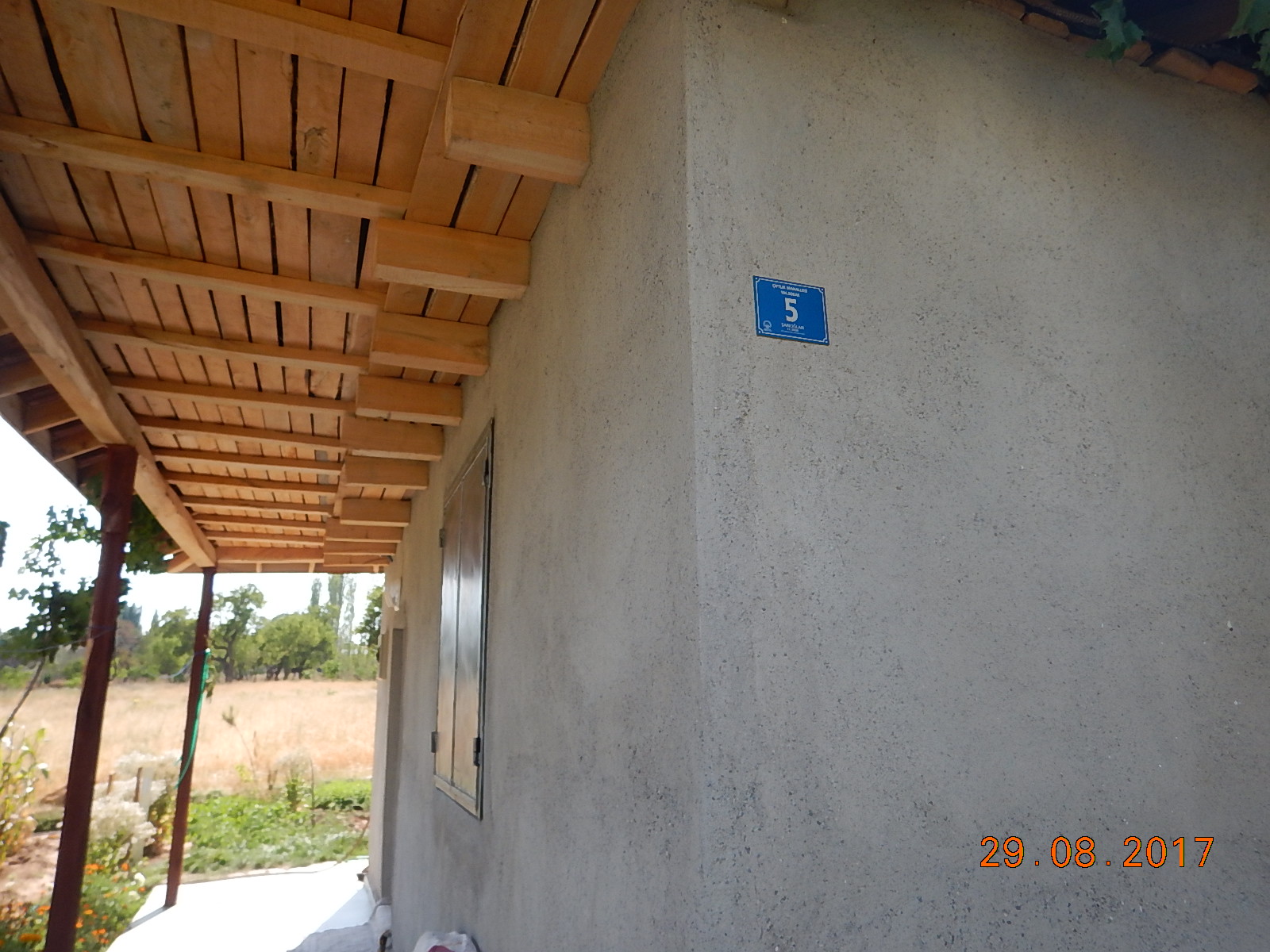

Supplement: Supplemental Information 2 [file peerj-cs-09-1453-s002.zip › ExampleDataFile/31xxyyyz5.JPG]

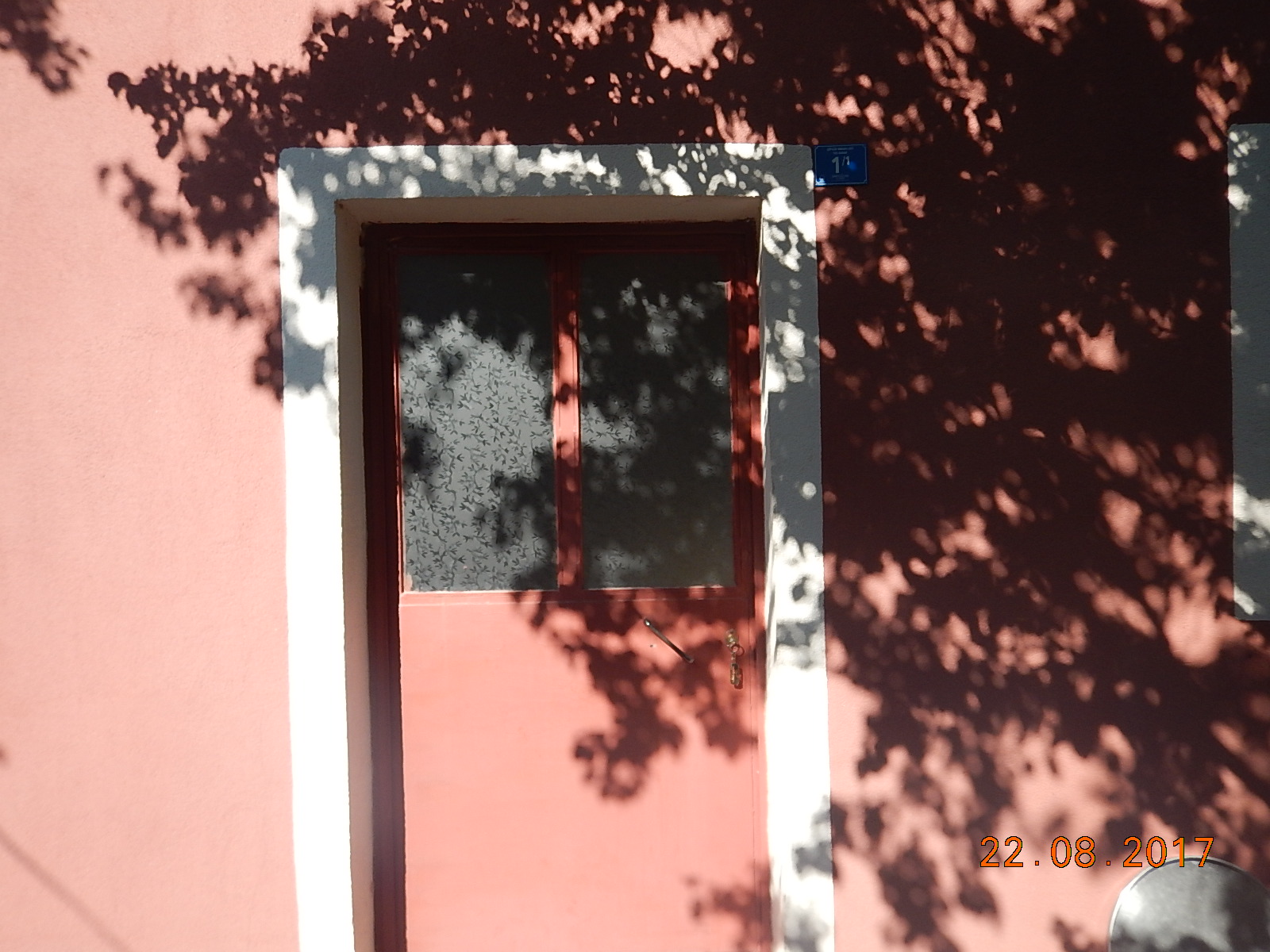

Supplement: Supplemental Information 2 [file peerj-cs-09-1453-s002.zip › ExampleDataFile/32xxyyyz1.1.JPG]

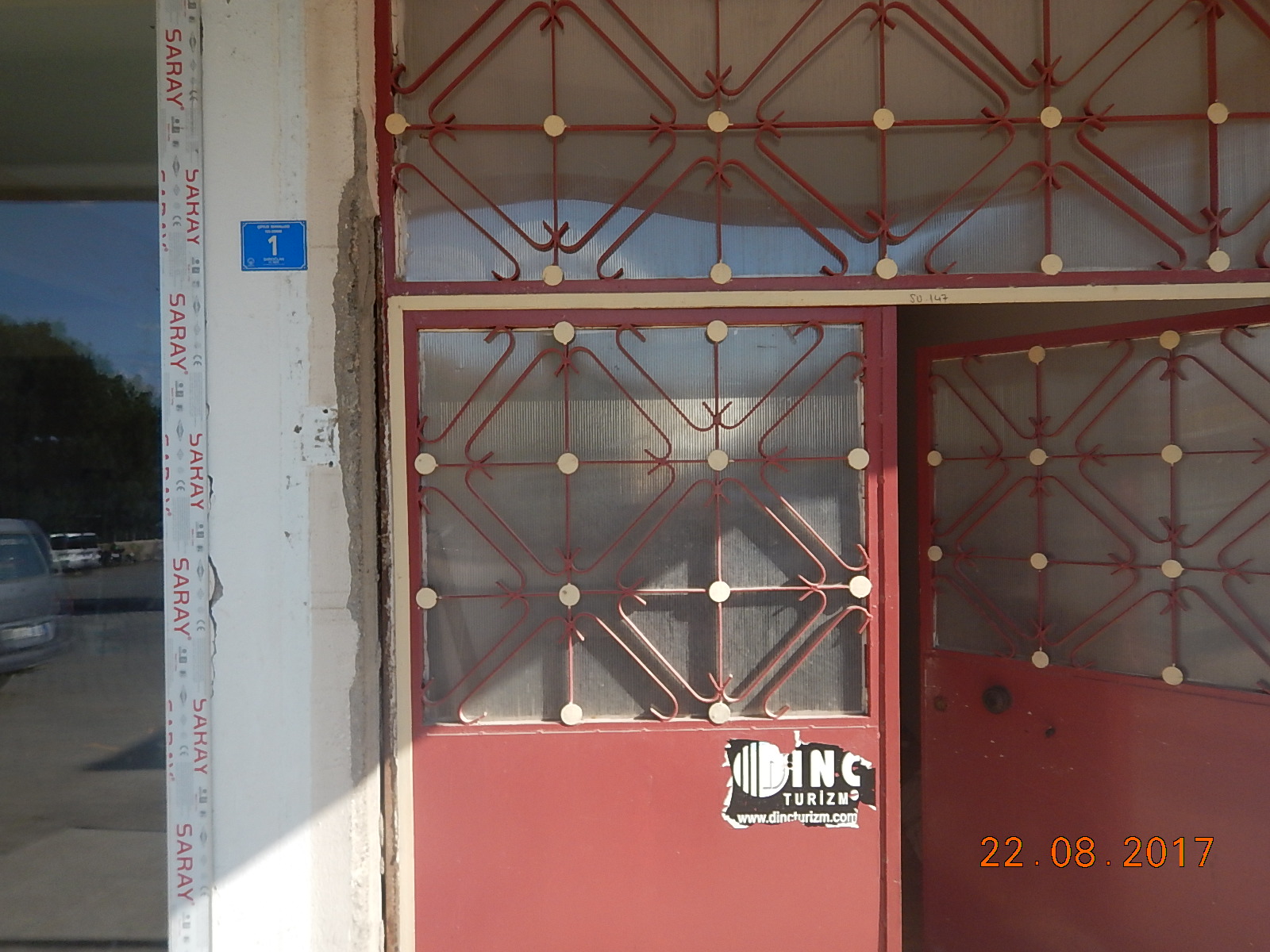

Supplement: Supplemental Information 2 [file peerj-cs-09-1453-s002.zip › ExampleDataFile/33xxyyyz1.JPG]

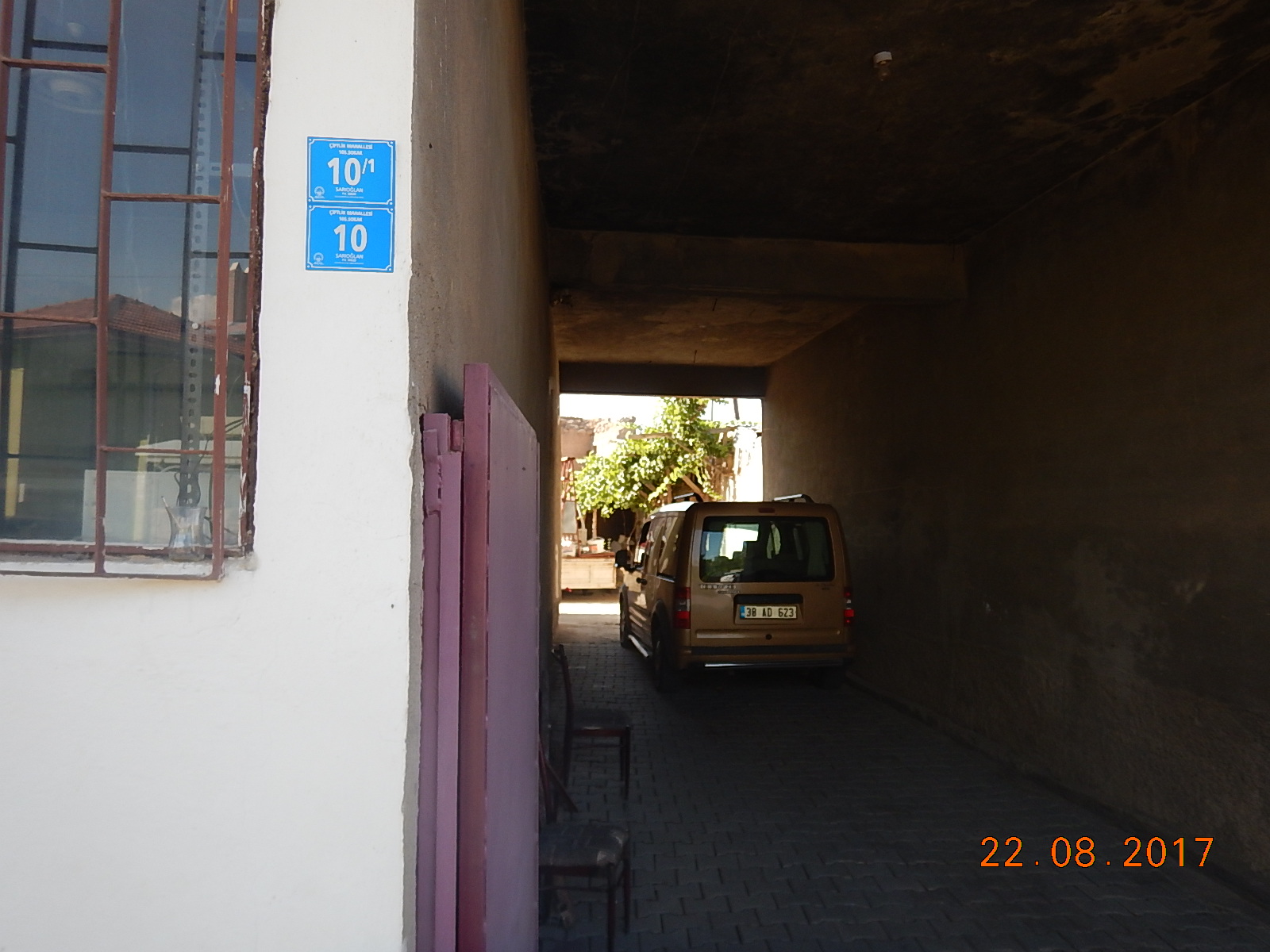

Supplement: Supplemental Information 2 [file peerj-cs-09-1453-s002.zip › ExampleDataFile/34xxyyyz10-10.1.JPG]

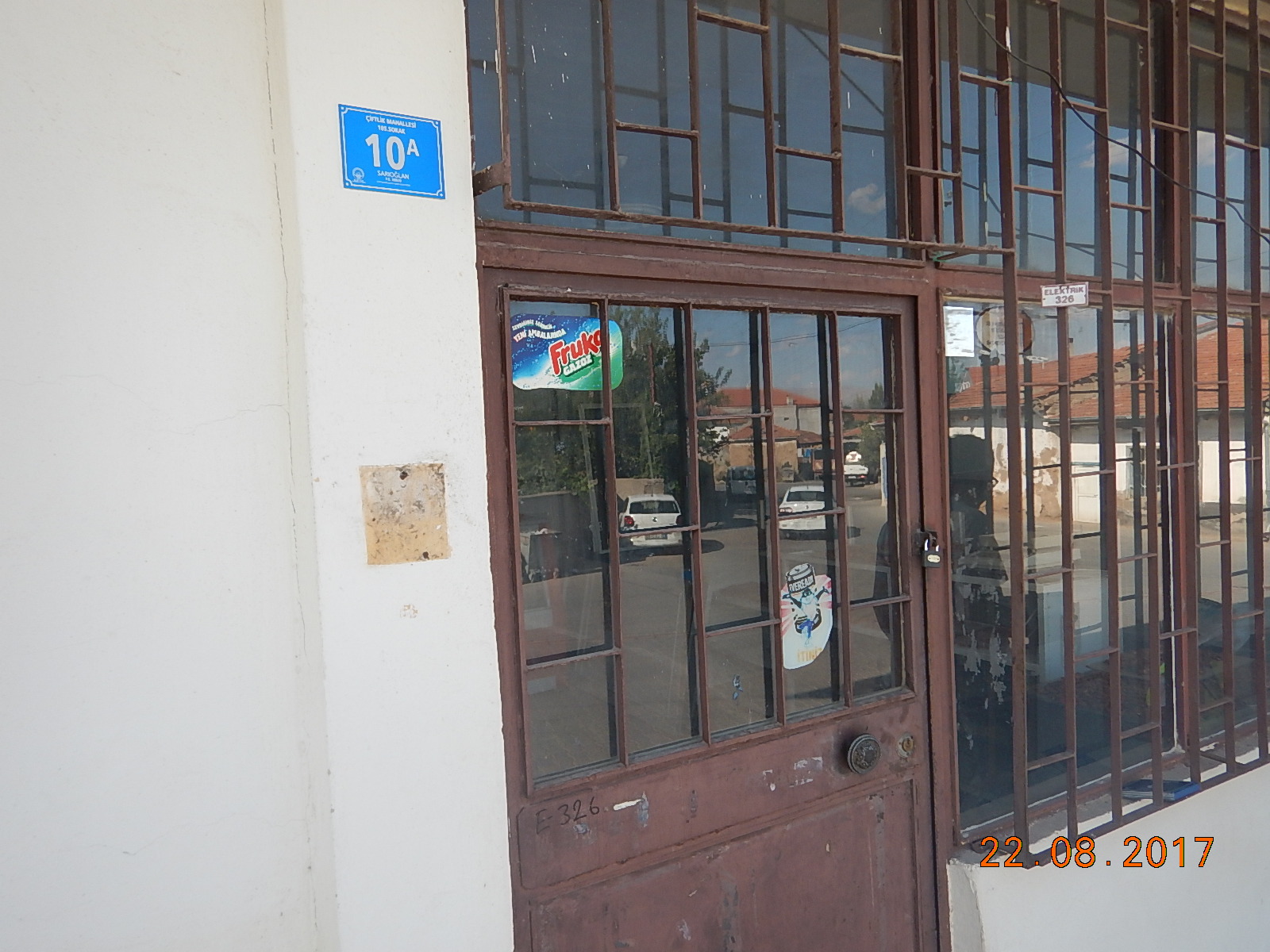

Supplement: Supplemental Information 2 [file peerj-cs-09-1453-s002.zip › ExampleDataFile/35xxyyyz10A.JPG]

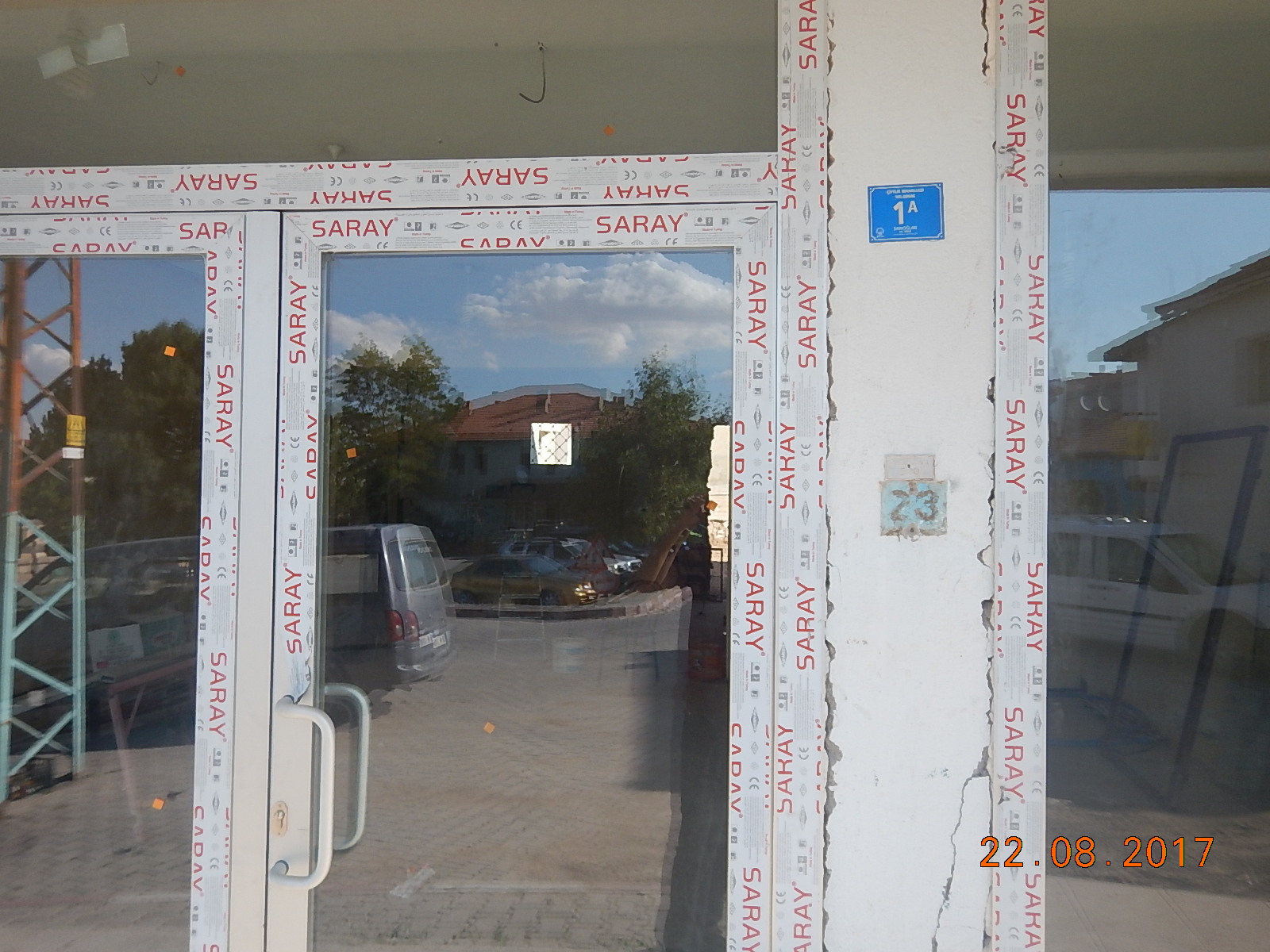

Supplement: Supplemental Information 2 [file peerj-cs-09-1453-s002.zip › ExampleDataFile/36xxyyyz1A.JPG]

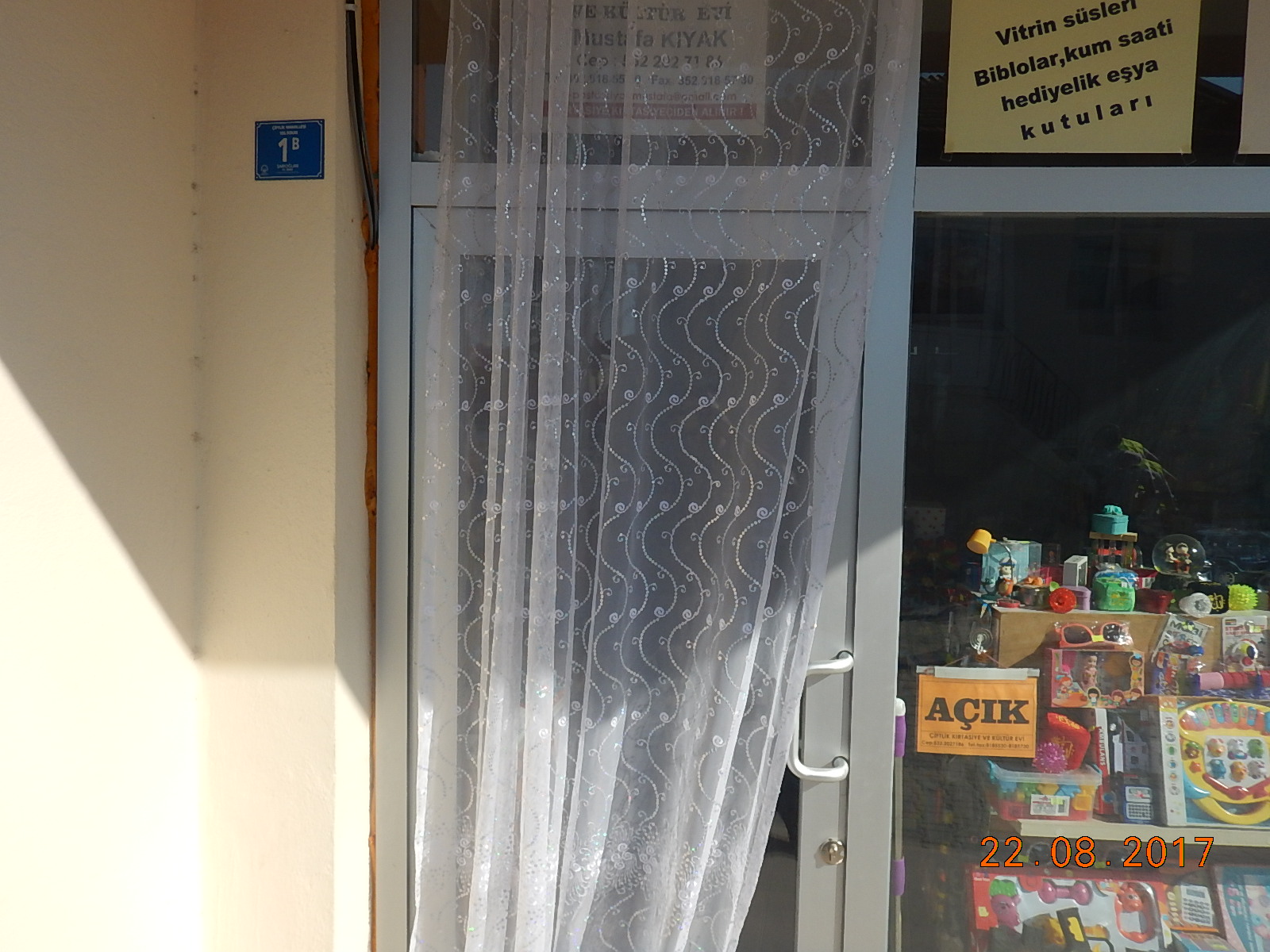

Supplement: Supplemental Information 2 [file peerj-cs-09-1453-s002.zip › ExampleDataFile/37xxyyyz1B.JPG]

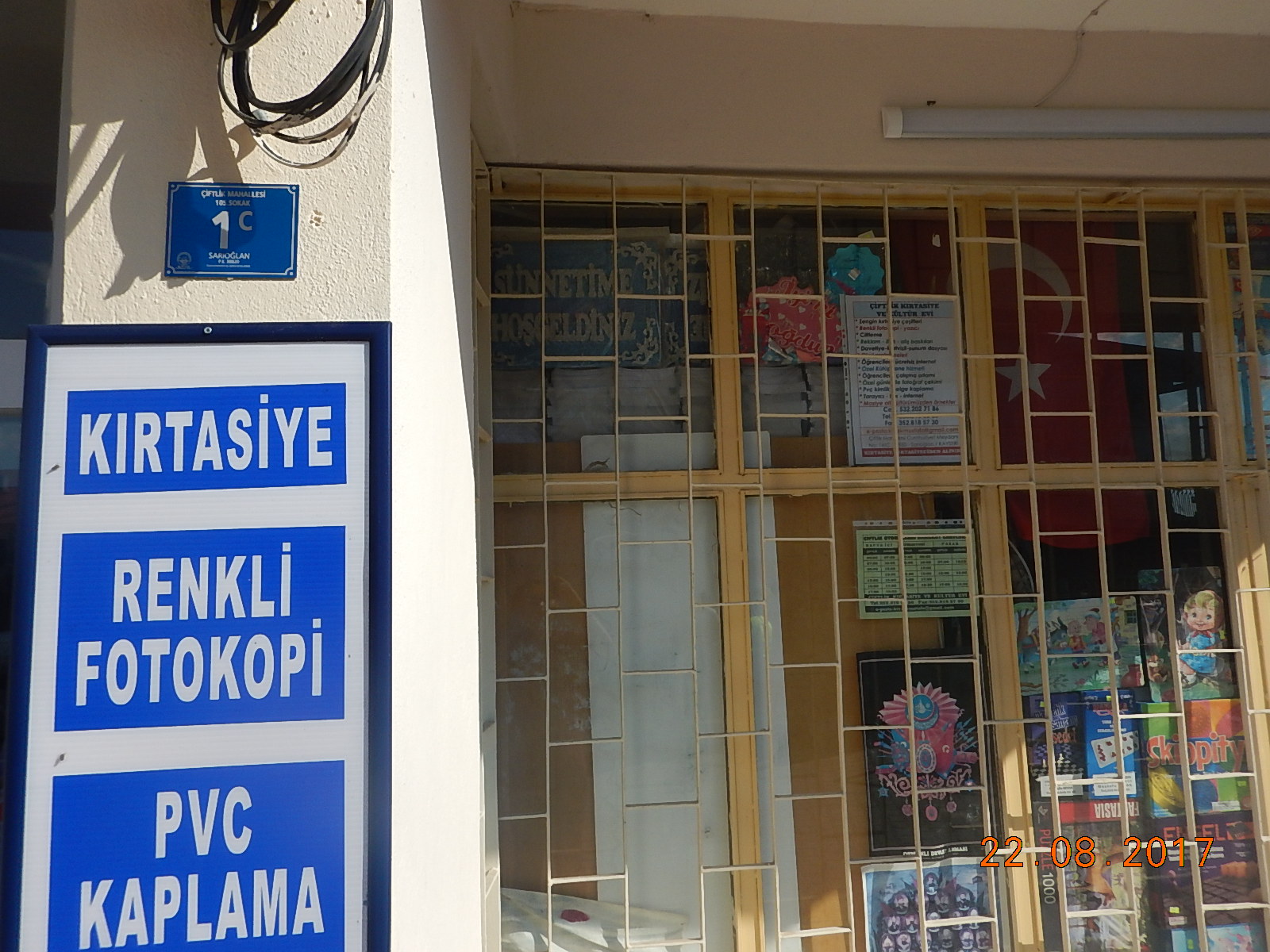

Supplement: Supplemental Information 2 [file peerj-cs-09-1453-s002.zip › ExampleDataFile/38xxyyyz1C.JPG]

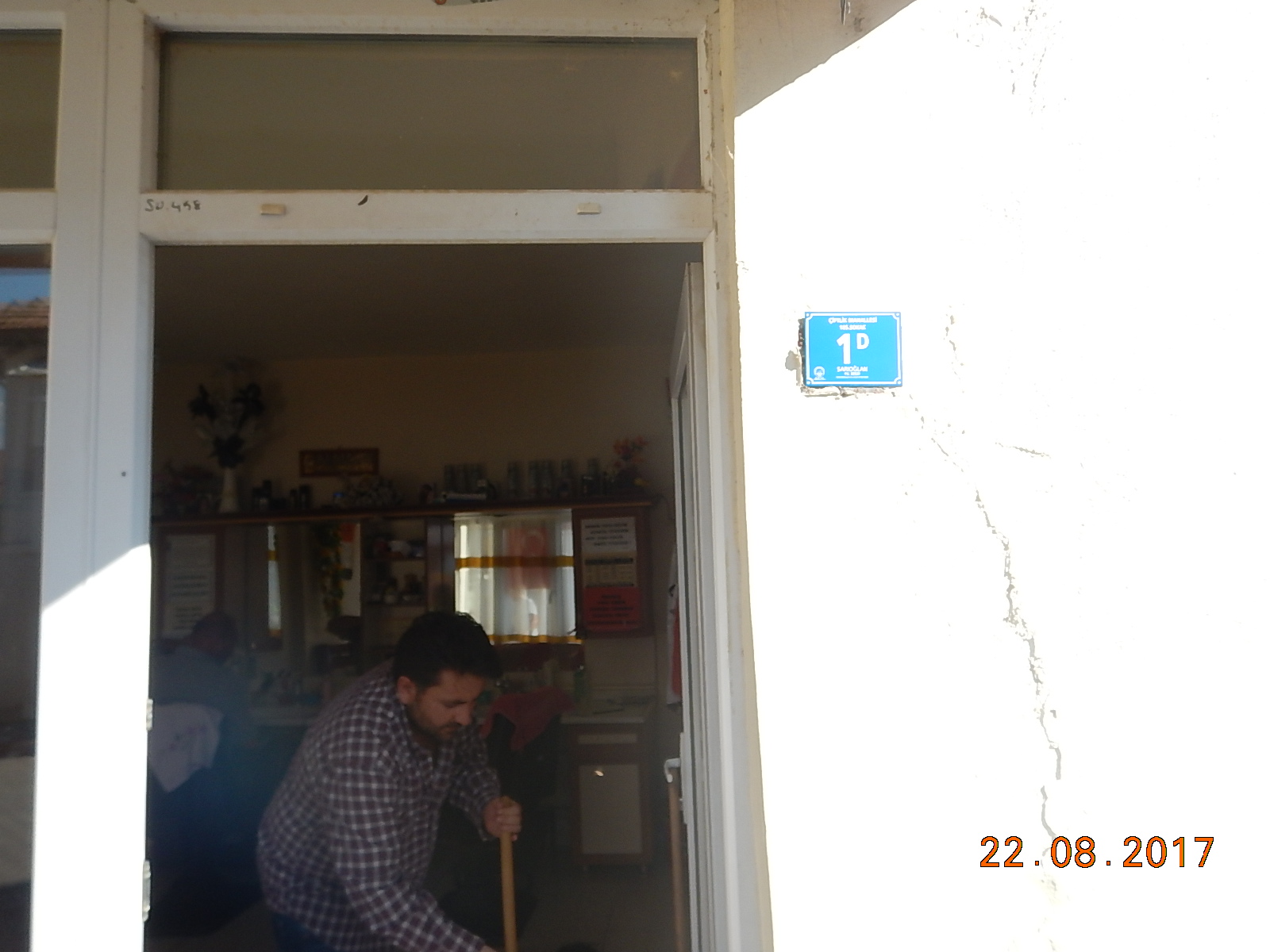

Supplement: Supplemental Information 2 [file peerj-cs-09-1453-s002.zip › ExampleDataFile/39xxyyyz1D.JPG]

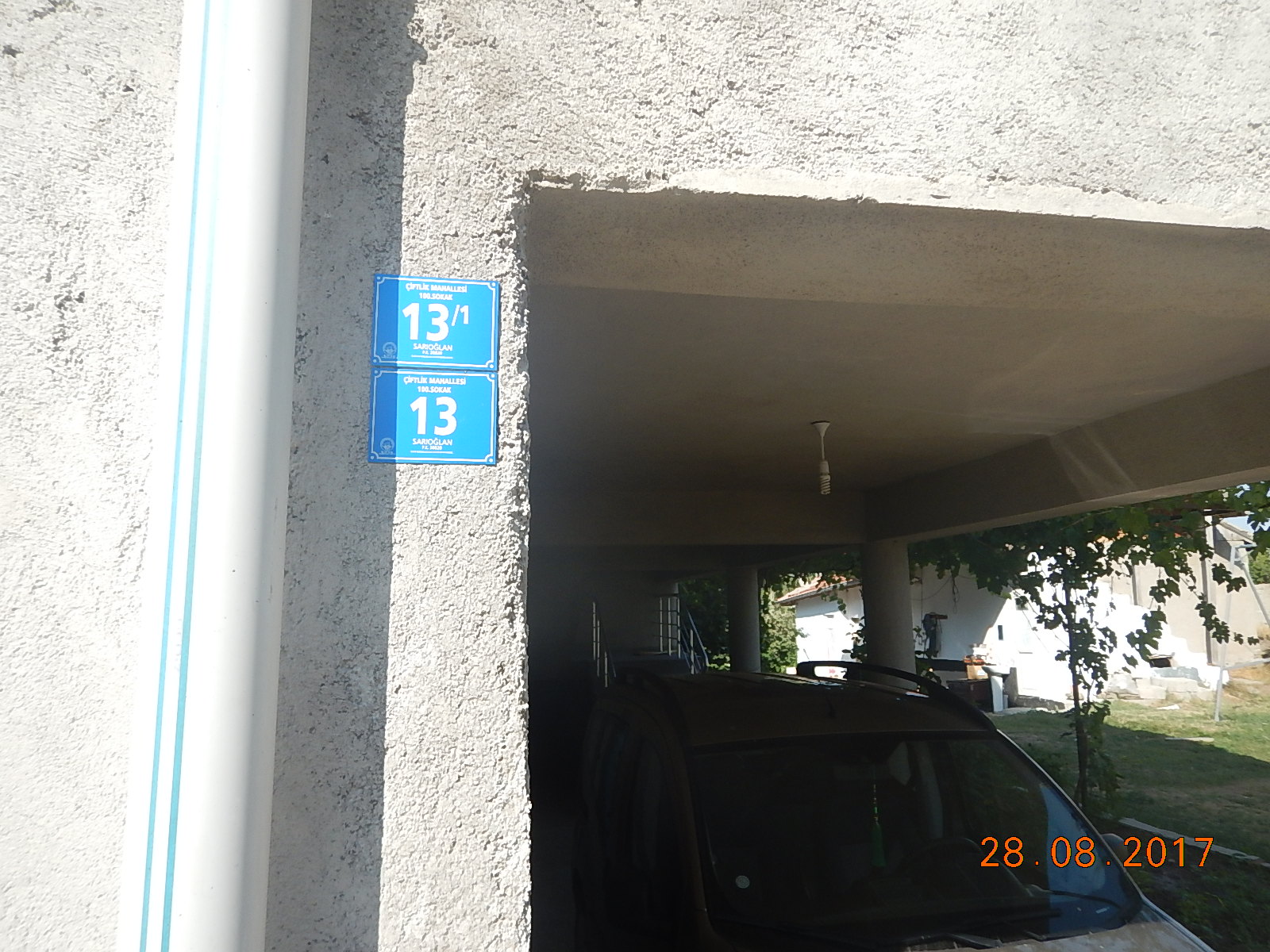

Supplement: Supplemental Information 2 [file peerj-cs-09-1453-s002.zip › ExampleDataFile/3xxyyyz13-13.1.JPG]

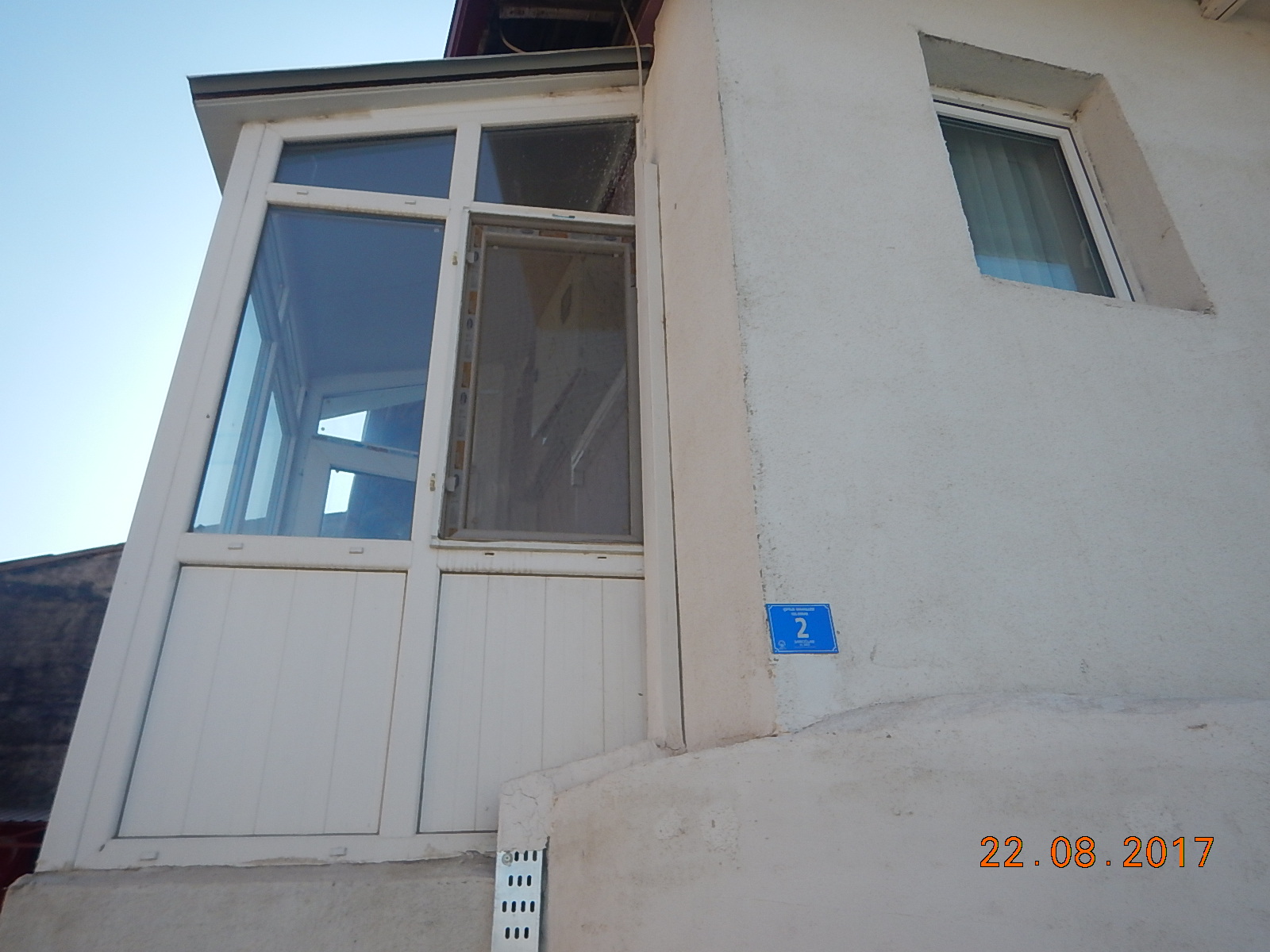

Supplement: Supplemental Information 2 [file peerj-cs-09-1453-s002.zip › ExampleDataFile/40xxyyyz2.JPG]

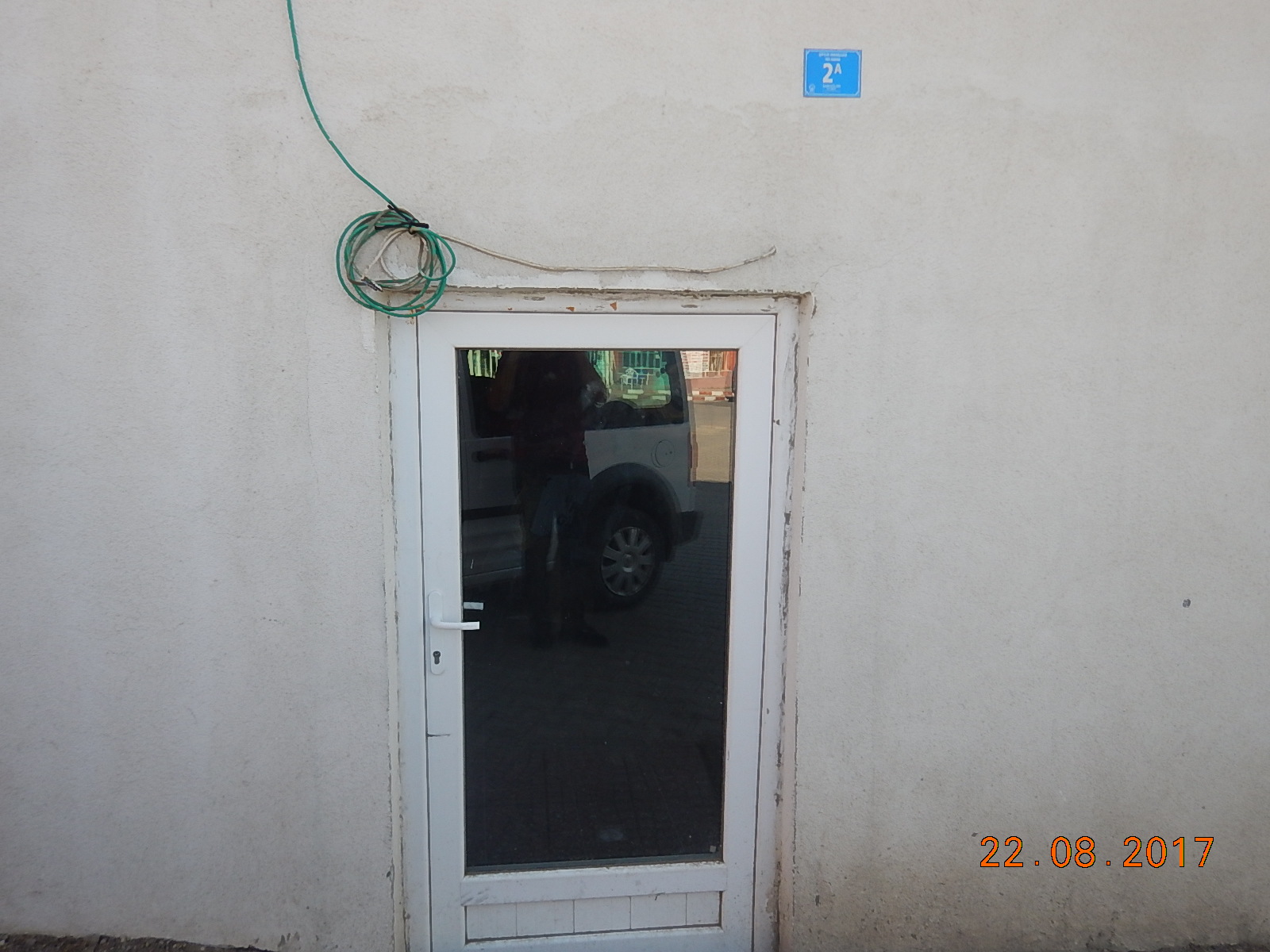

Supplement: Supplemental Information 2 [file peerj-cs-09-1453-s002.zip › ExampleDataFile/41xxyyyz2A.JPG]

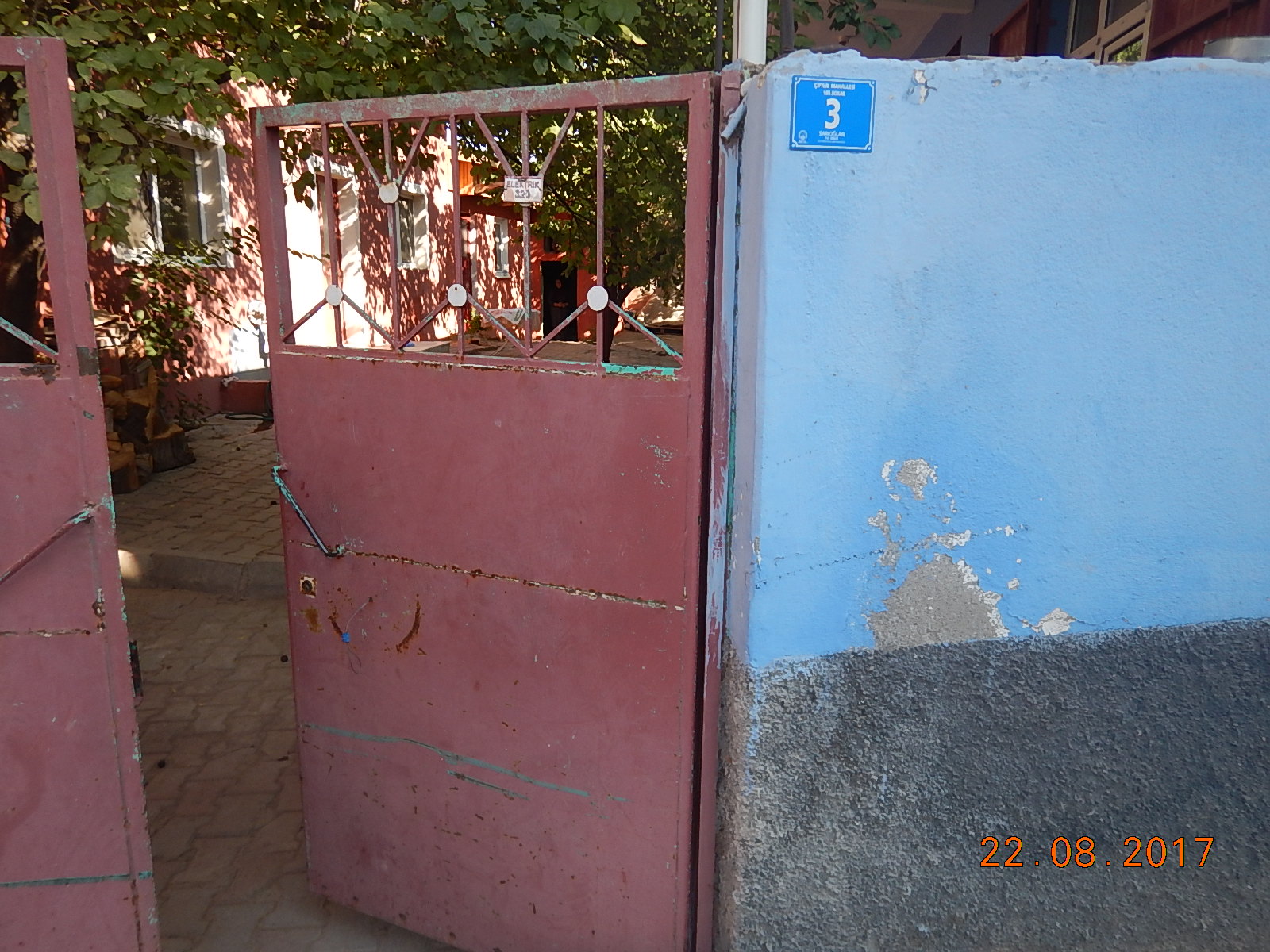

Supplement: Supplemental Information 2 [file peerj-cs-09-1453-s002.zip › ExampleDataFile/42xxyyyz3.JPG]

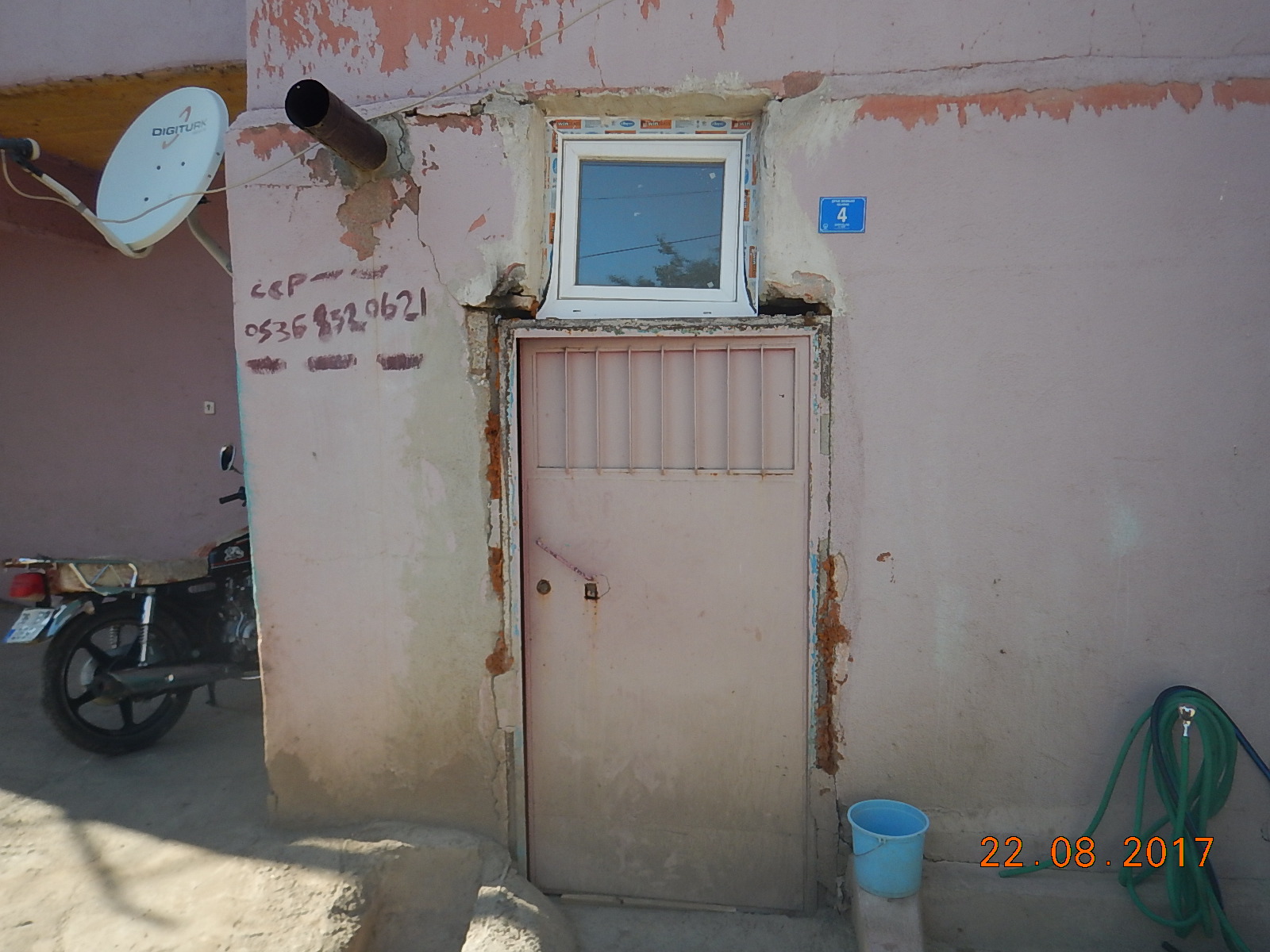

Supplement: Supplemental Information 2 [file peerj-cs-09-1453-s002.zip › ExampleDataFile/43xxyyyz4.JPG]

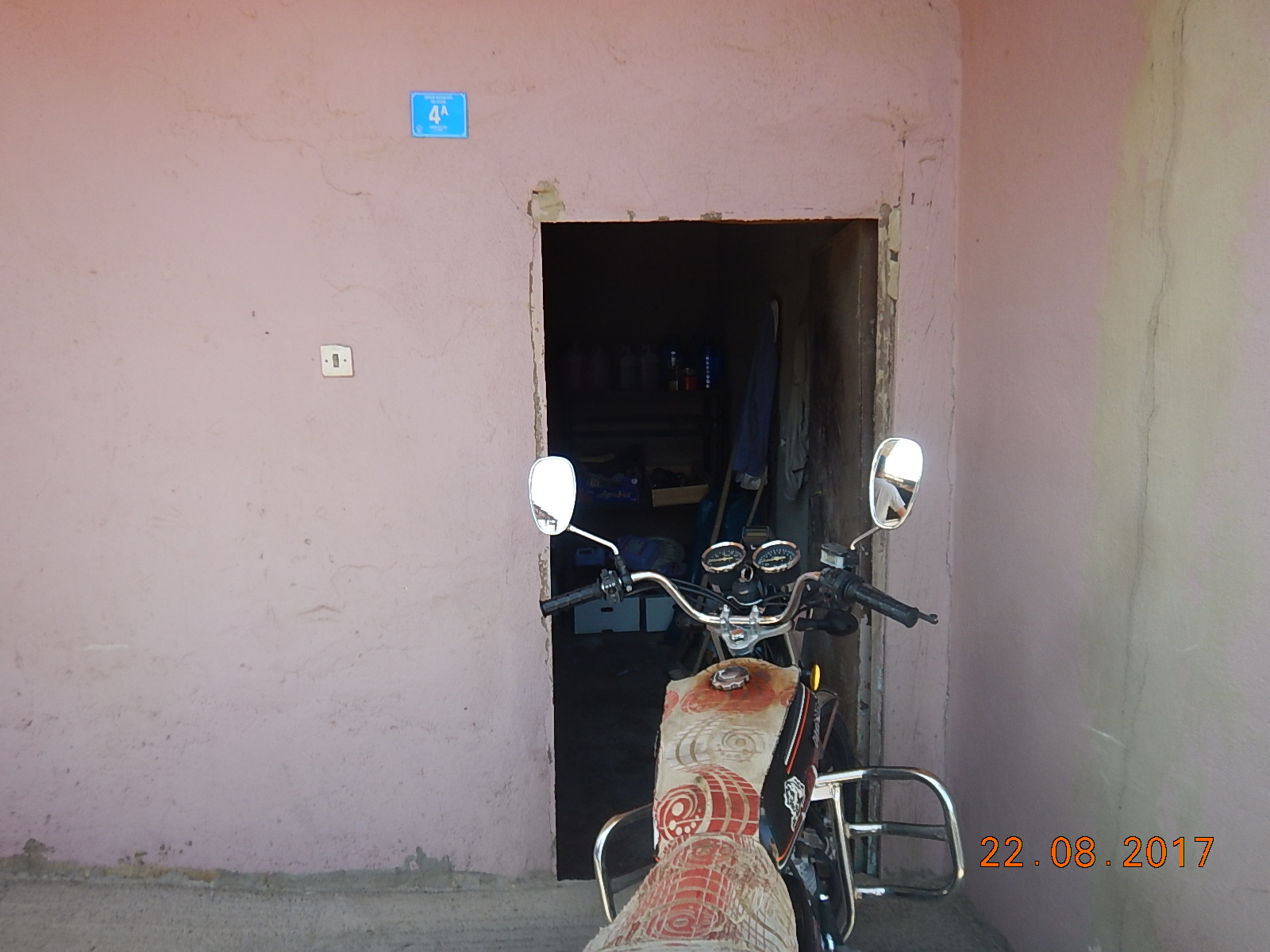

Supplement: Supplemental Information 2 [file peerj-cs-09-1453-s002.zip › ExampleDataFile/44xxyyyz4A.JPG]

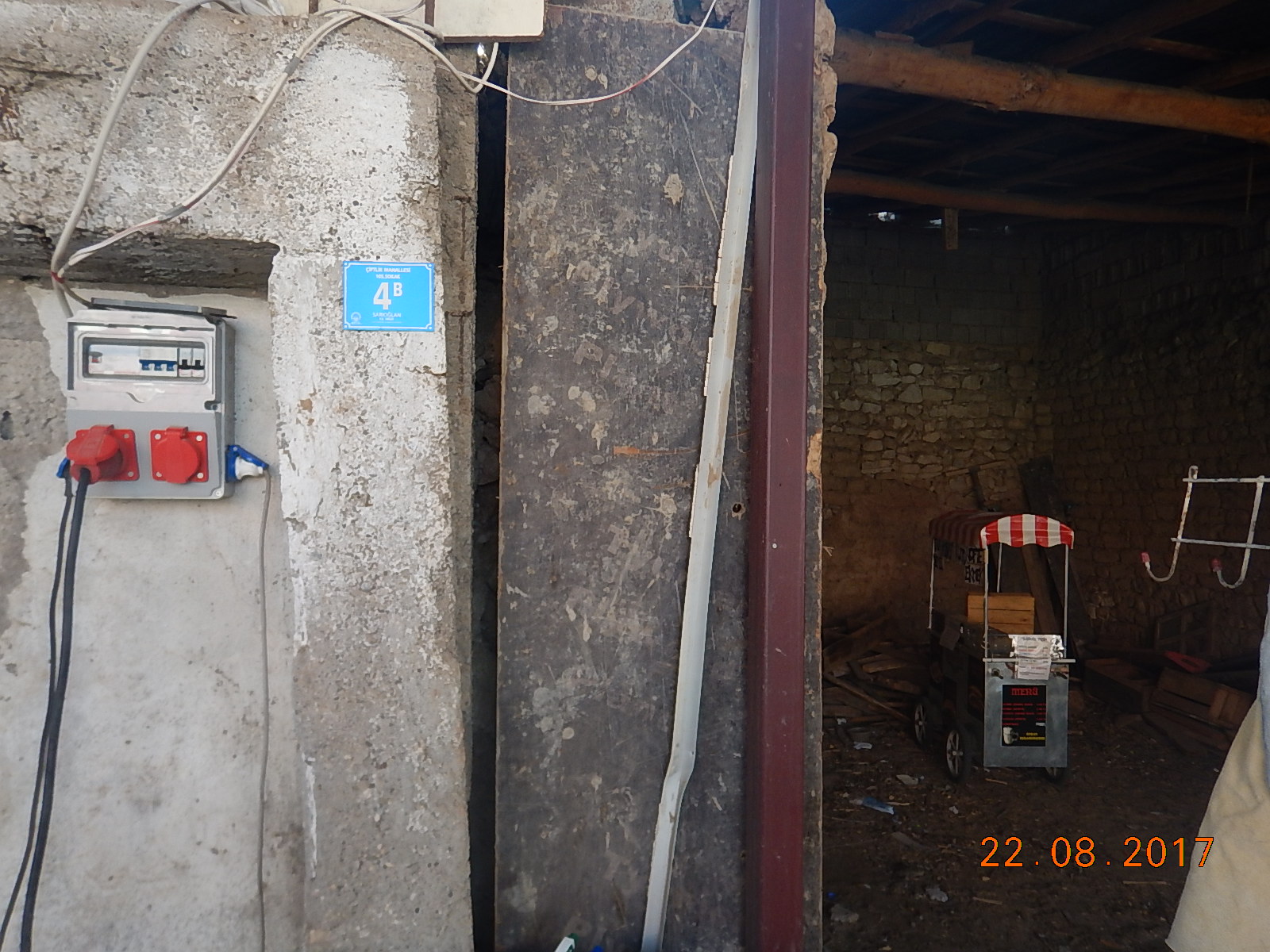

Supplement: Supplemental Information 2 [file peerj-cs-09-1453-s002.zip › ExampleDataFile/45xxyyyz4B.JPG]

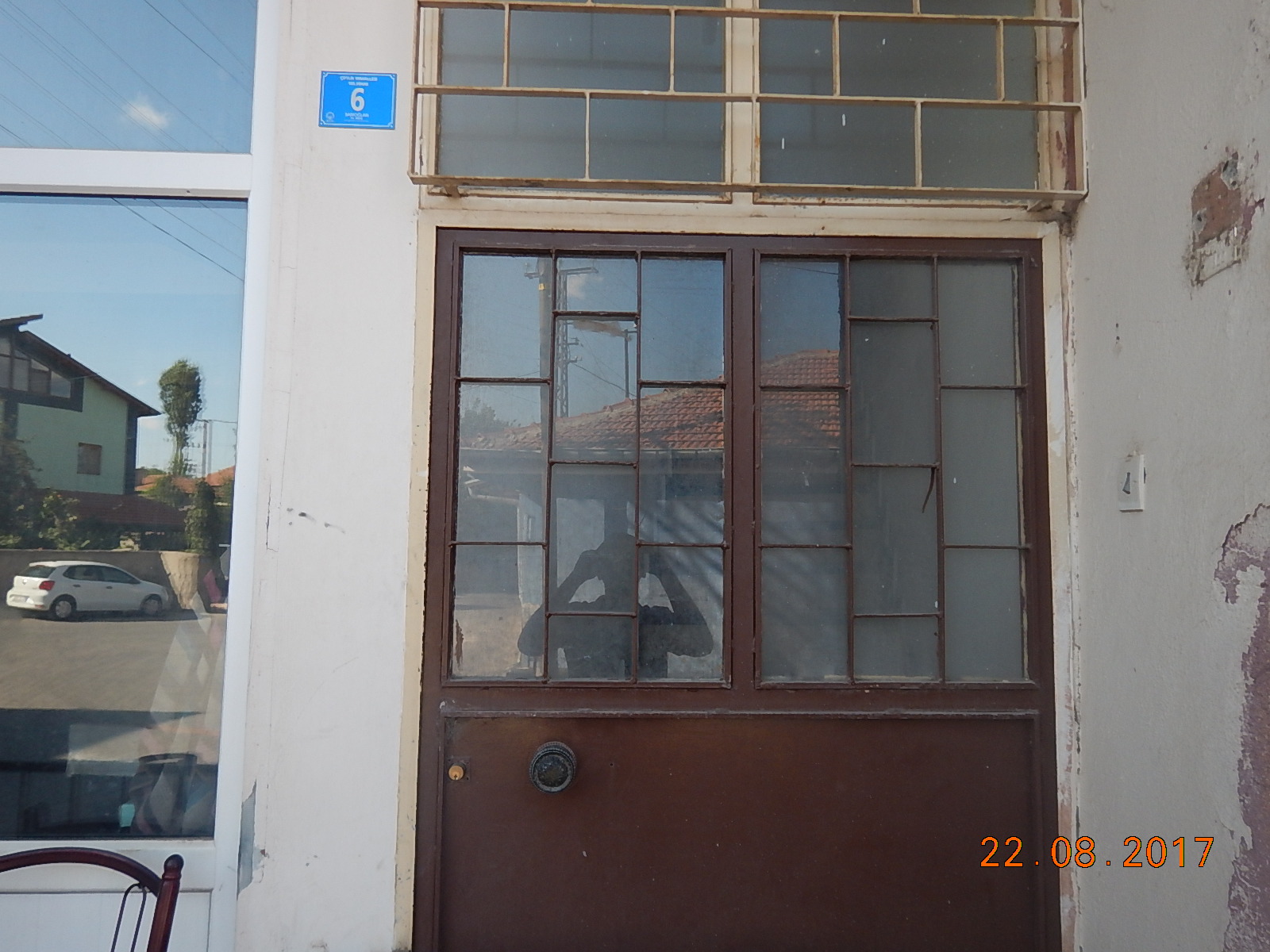

Supplement: Supplemental Information 2 [file peerj-cs-09-1453-s002.zip › ExampleDataFile/46xxyyyz6.JPG]

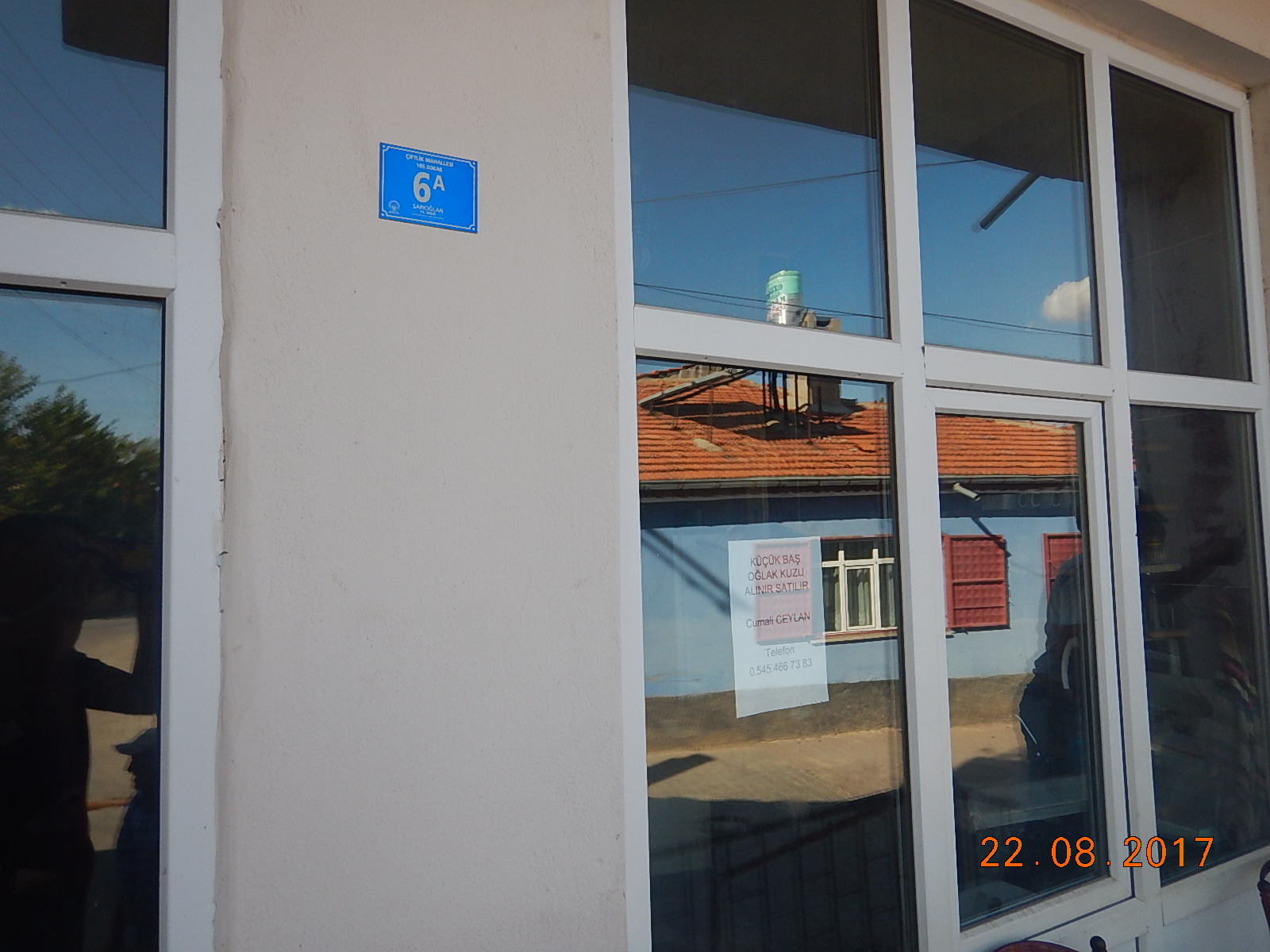

Supplement: Supplemental Information 2 [file peerj-cs-09-1453-s002.zip › ExampleDataFile/47xxyyyz6A.JPG]

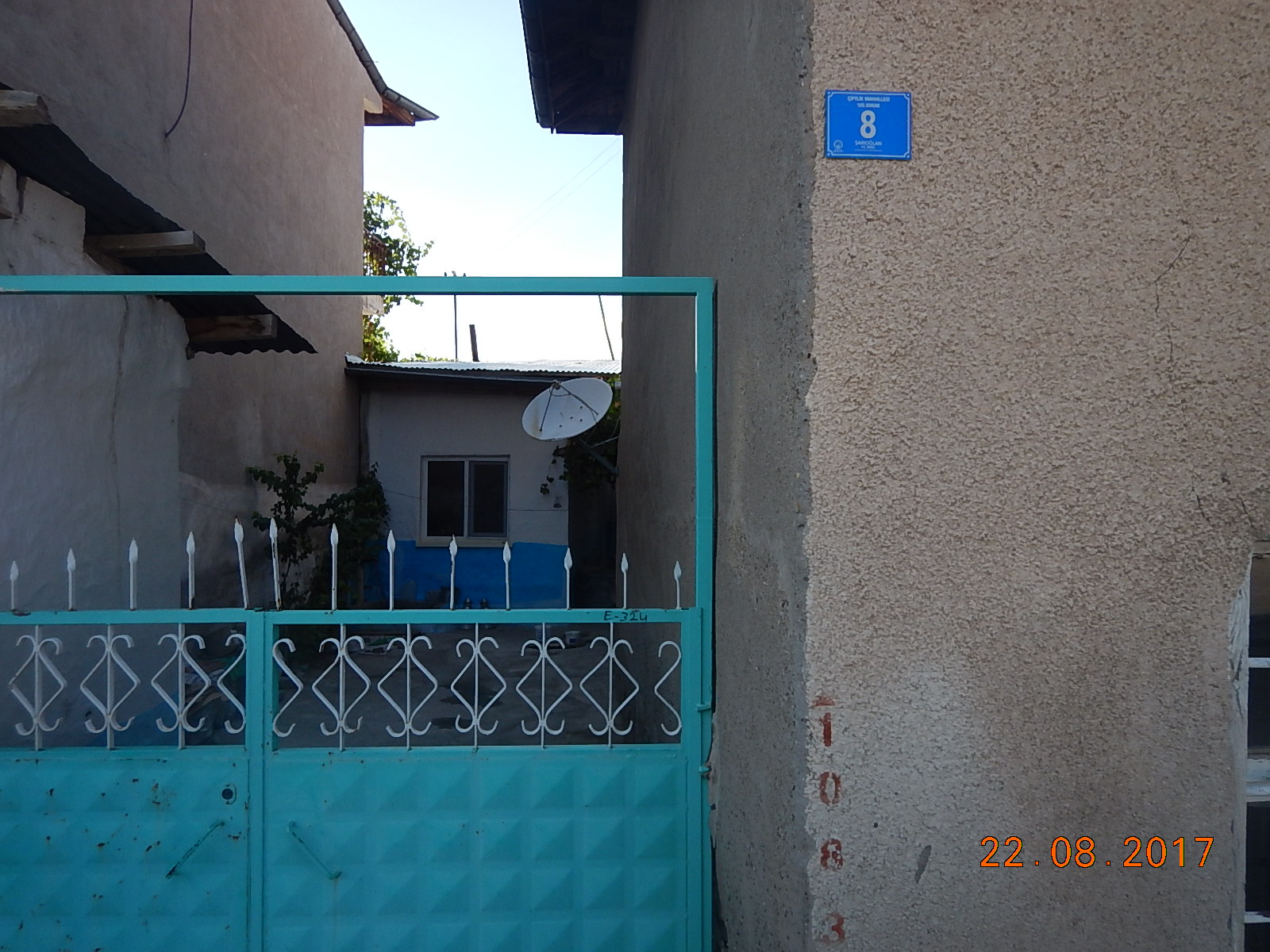

Supplement: Supplemental Information 2 [file peerj-cs-09-1453-s002.zip › ExampleDataFile/48xxyyyz8.JPG]

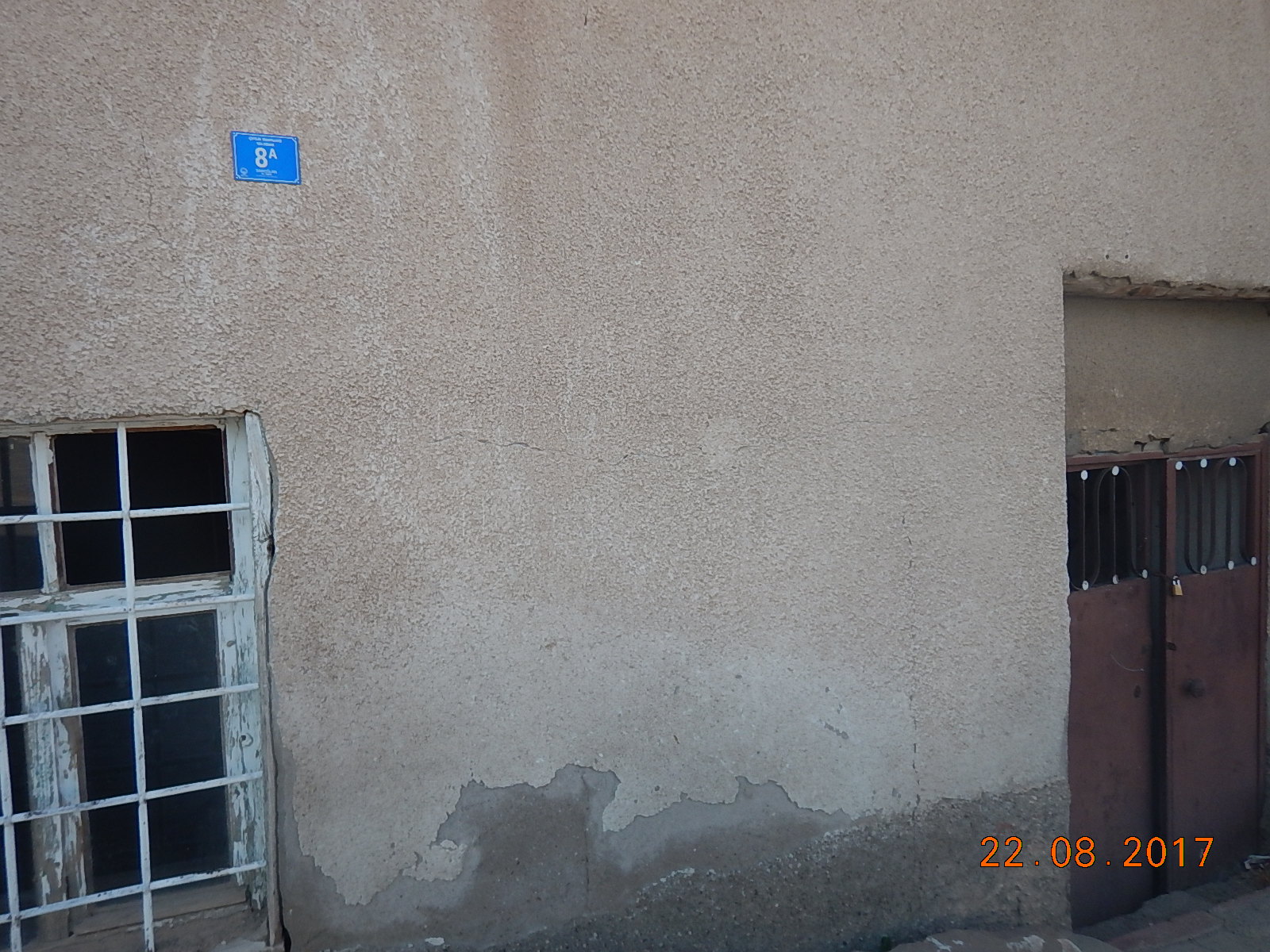

Supplement: Supplemental Information 2 [file peerj-cs-09-1453-s002.zip › ExampleDataFile/49xxyyyz8A.JPG]

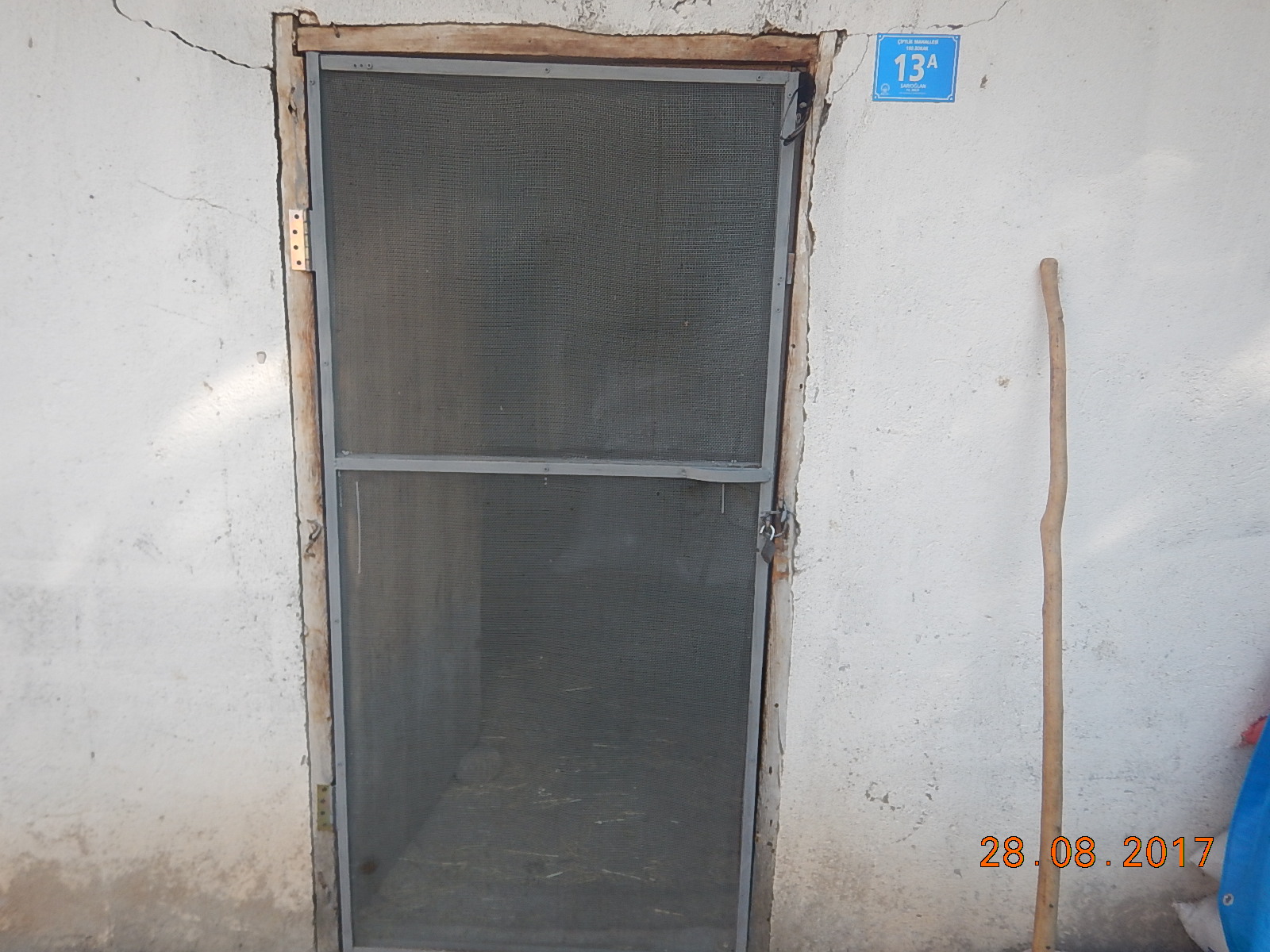

Supplement: Supplemental Information 2 [file peerj-cs-09-1453-s002.zip › ExampleDataFile/4xxyyyz13A.JPG]

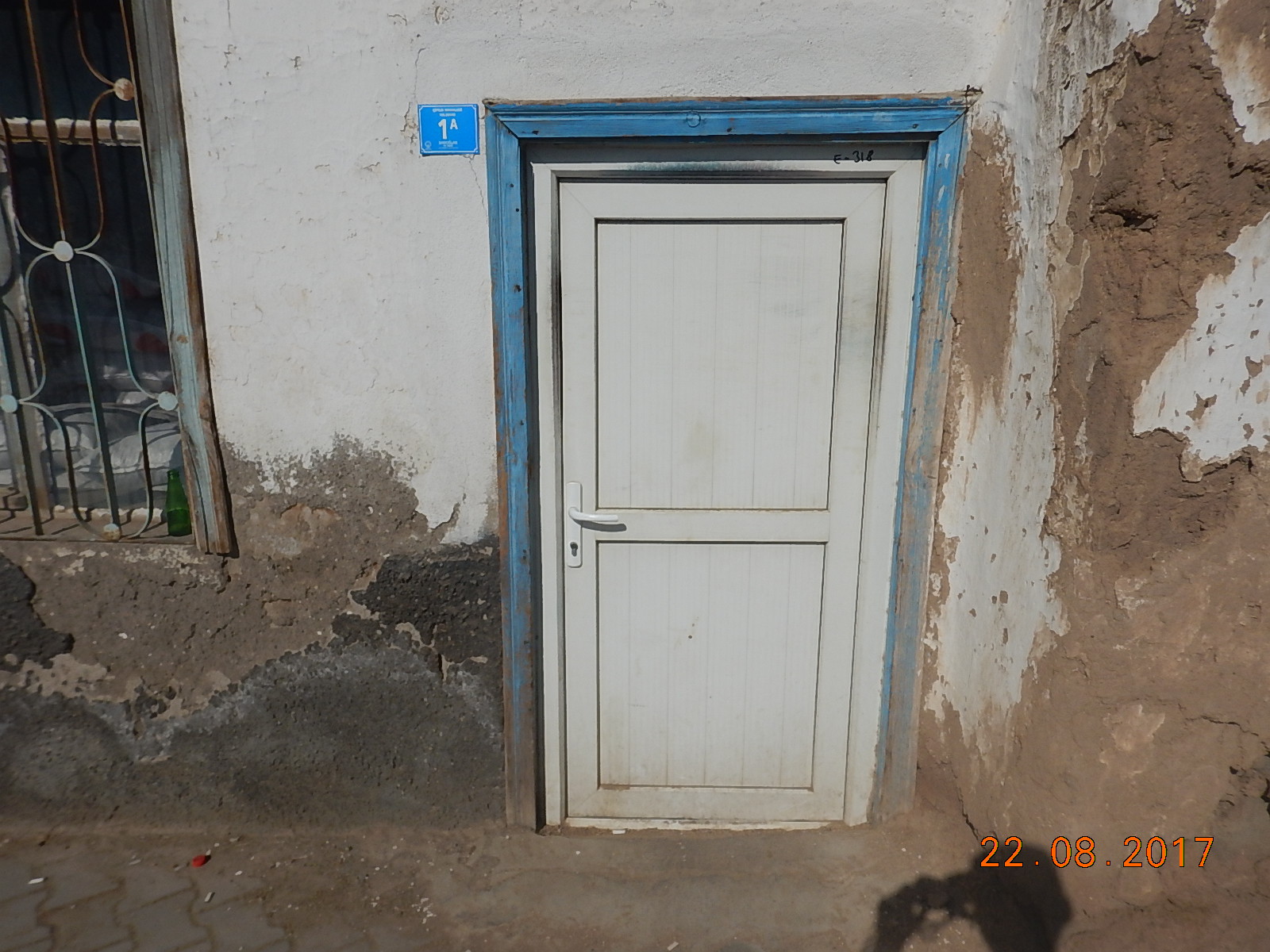

Supplement: Supplemental Information 2 [file peerj-cs-09-1453-s002.zip › ExampleDataFile/50xxyyyz1A.JPG]

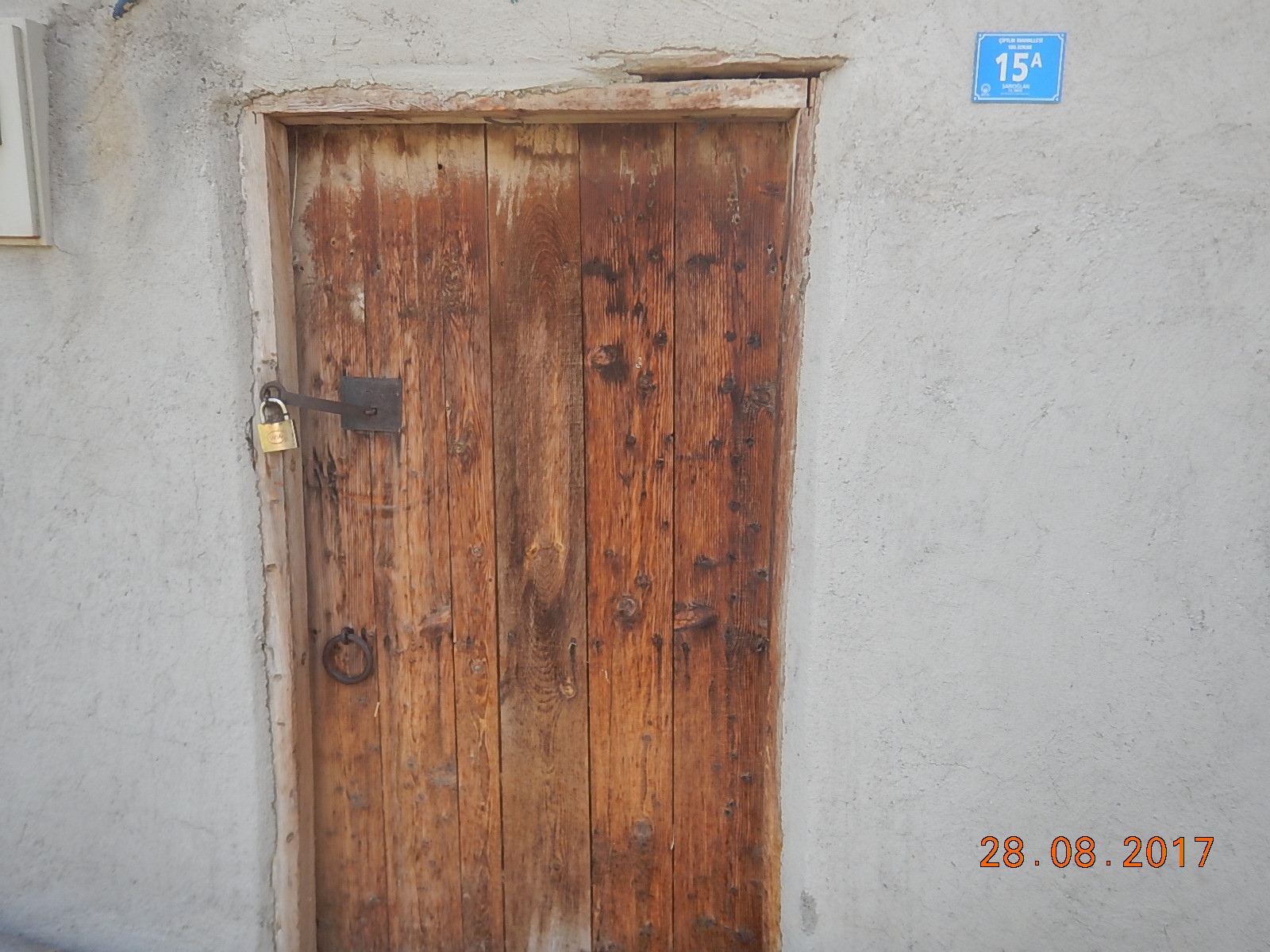

Supplement: Supplemental Information 2 [file peerj-cs-09-1453-s002.zip › ExampleDataFile/5xxyyyz15A.JPG]

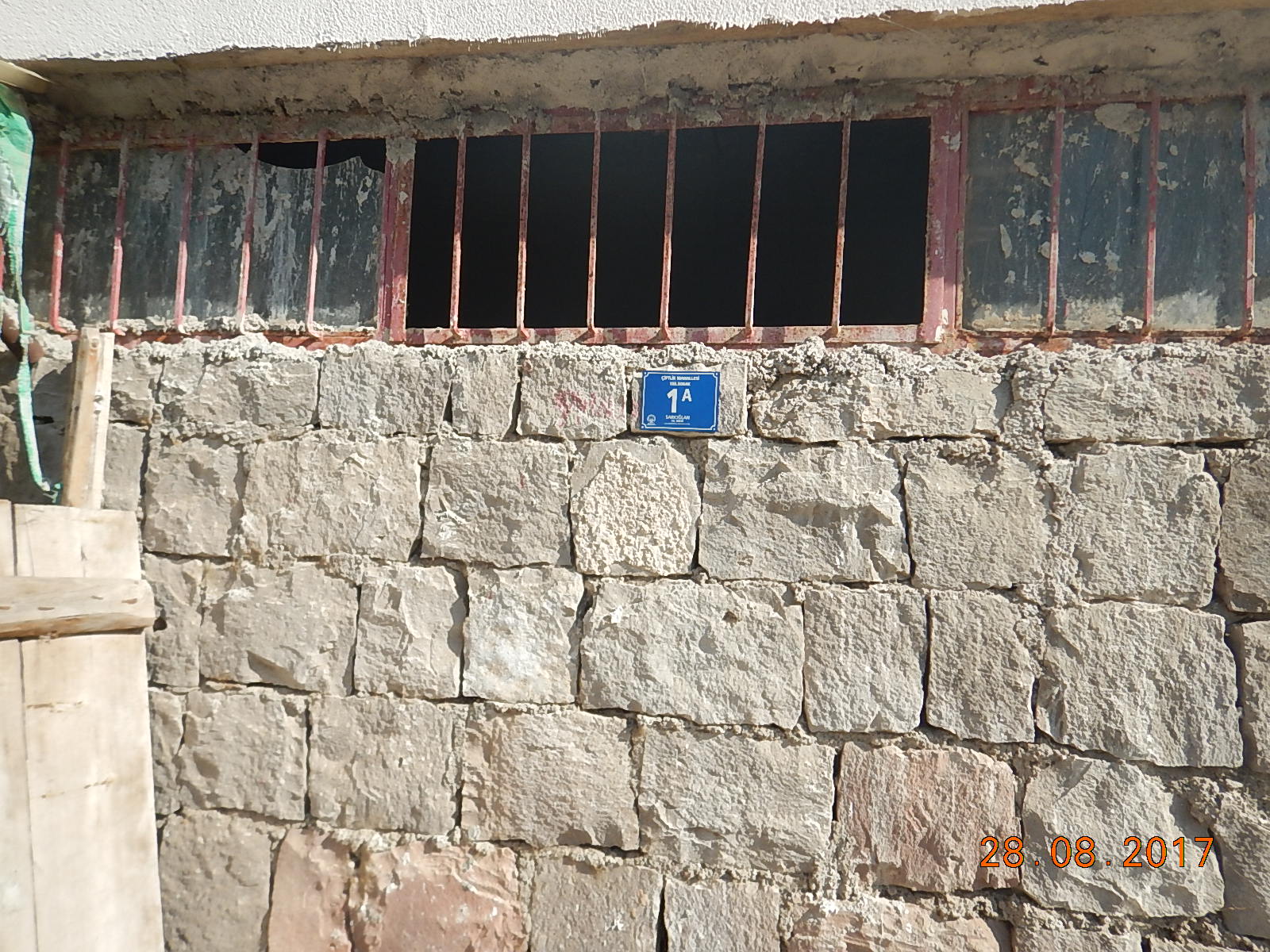

Supplement: Supplemental Information 2 [file peerj-cs-09-1453-s002.zip › ExampleDataFile/6xxyyyz1A.JPG]

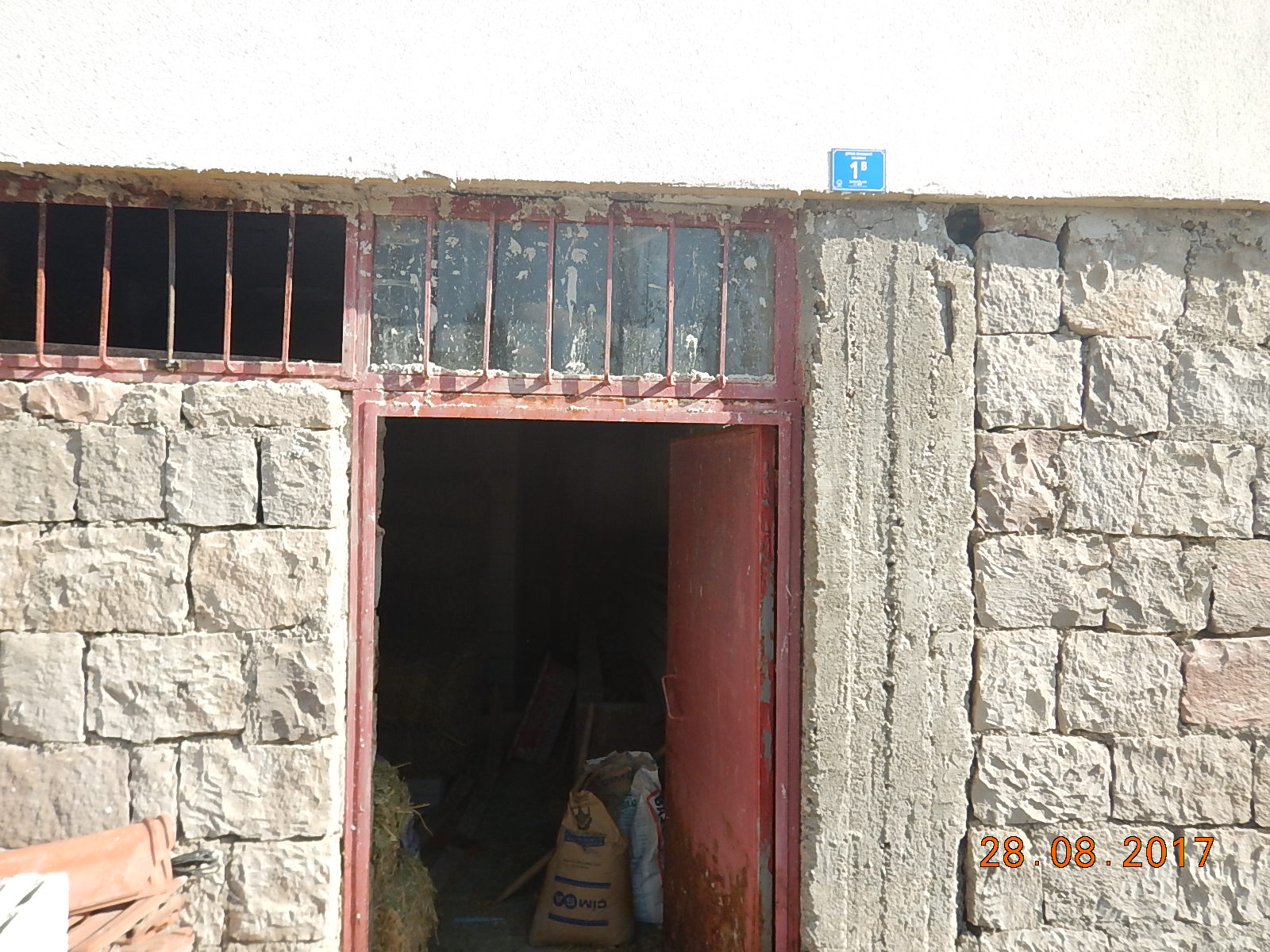

Supplement: Supplemental Information 2 [file peerj-cs-09-1453-s002.zip › ExampleDataFile/7xxyyyz1B.JPG]

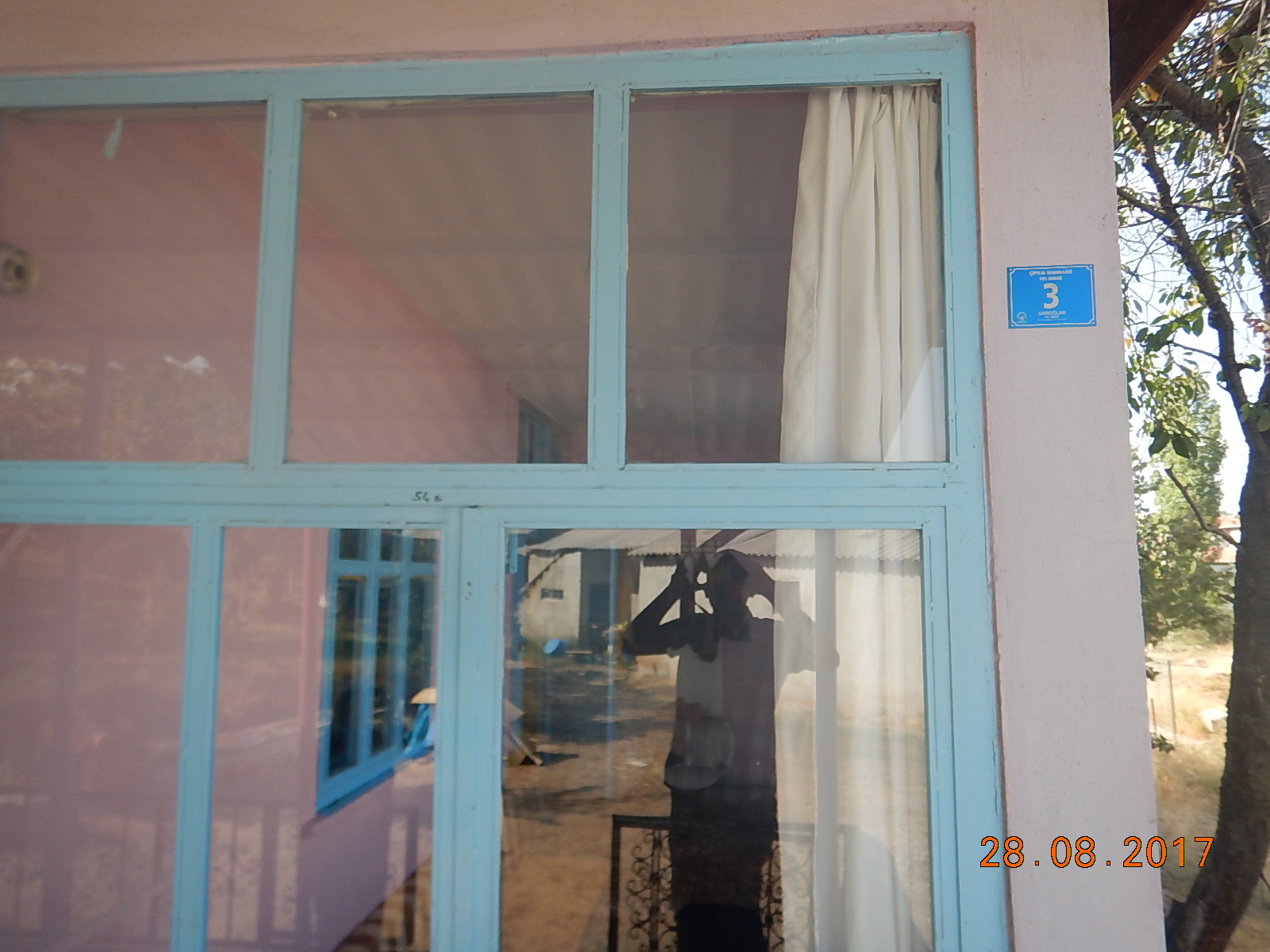

Supplement: Supplemental Information 2 [file peerj-cs-09-1453-s002.zip › ExampleDataFile/8xxyyyz3.JPG]

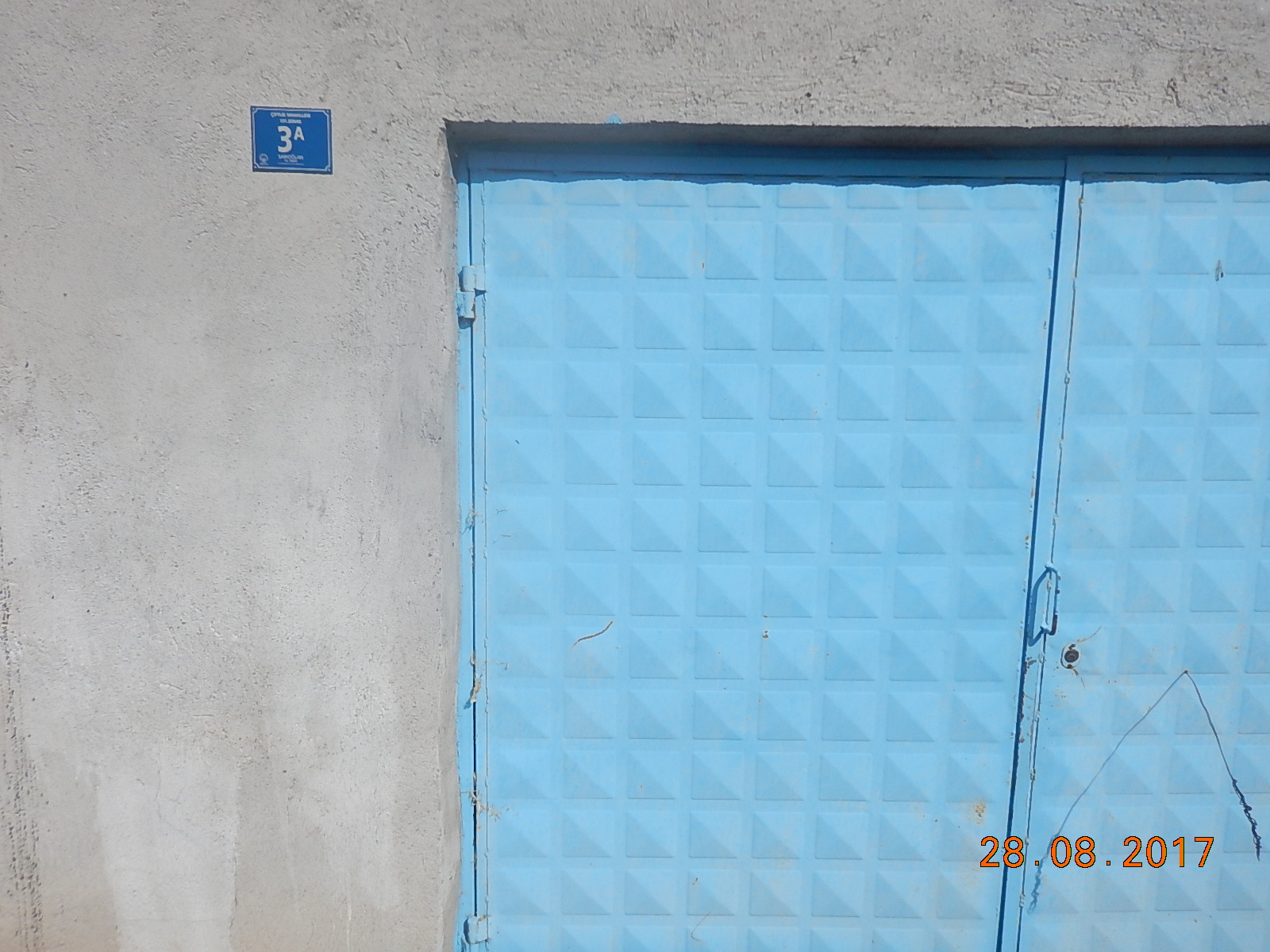

Supplement: Supplemental Information 2 [file peerj-cs-09-1453-s002.zip › ExampleDataFile/9xxyyyz3A.JPG]
